# Machine Learning-Based Estimation of Mechanical Properties of 3D-Printed PLA Composites Under Annealing Treatment

**DOI:** 10.3390/polym18151808

**Published:** 2026-07-23

**Authors:** Ahmed Fouly

**Affiliations:** Department of Mechanical Engineering, College of Engineering, King Saud University, P.O. Box 800, Riyadh 11421, Saudi Arabia; amohammed7.c@ksu.edu.sa

**Keywords:** PLA green composite, sustainable materials, waste valorization, Gaussian process regression, biomedical composites, annealing treatment

## Abstract

This study presents a novel, data-driven framework for predicting and optimizing the mechanical performance of 3D-printed polylactic acid (PLA) composites reinforced with date pit (DP) particles under controlled annealing conditions. This work uniquely integrates both effects into a unified machine learning (ML) surrogate modeling approach, enabling simultaneous property prediction and design optimization from a minimal experimental dataset. Three ML algorithms, Gaussian Process Regression (GPR), Random Forest Regression (RFR), and Decision Tree Regression (DTR), were systematically developed to predict Young’s modulus (E), ultimate compressive strength (UCS), and Shore D hardness as functions of DP weight fraction (0–10 wt.%) and annealing duration (0–20 h at 100 °C). Among the models, GPR demonstrated superior generalization and uncertainty-aware prediction capability, achieving test R^2^ values up to 0.936 and a MAPE below 2% for structurally critical properties. Beyond prediction accuracy, the study introduces a key novelty through the integration of Partial Dependence Plot (PDP) analysis, which reveals physically interpretable relationships between processing parameters and mechanical behavior. A non-monotonic annealing optimum at 5 h was identified, governed by competing crystallization enhancement and thermal degradation mechanisms, alongside a consistently positive reinforcement effect of DP loading. The optimized condition (10 wt.% DP, 5 h annealing) yielded improvements of 37.5% in stiffness, 42.4% in strength, and 21.6% in hardness compared to neat PLA. A five-fold cross-validation and split ratio sensitivity analyses (70/30, 80/20, 90/10) were conducted to assess generalization reliability. One-way ANOVA confirmed highly significant effects of DP loading on all three properties (*p* < 0.001 for E and hardness; *p* < 0.01 for UCS) and a significant effect of annealing time on UCS (*p* < 0.01). Permutation feature importance analysis confirmed DP weight fraction as the dominant predictor for all three outputs.

## 1. Introduction

The global healthcare sector faces mounting pressure to develop patient-specific orthotics and rehabilitation devices that are simultaneously affordable, customizable, and environmentally benign. Conventional orthotics fabricated from aluminum alloys, carbon fiber composites, or thermoformed polyolefins are effective but expensive to produce in bespoke geometries, with commercial knee braces retailing at $100–$900 per unit [1]. Fused deposition modeling (FDM) offers a compelling alternative: it enables rapid, low-cost fabrication of complex anatomical geometries directly from digital models without tooling or molds and has been successfully applied to produce prosthetic sockets, ankle-foot orthoses, and wrist splints [2,3]. The dominant FDM feedstock for biomedical applications is polylactic acid (PLA), a semi-crystalline biopolymer derived from fermented corn starch that combines biocompatibility, biodegradability, a low warping tendency, and ease of processing into 1.75 mm filaments suitable for desktop printers [4,5]. However, the mechanical performance of neat 3D-printed PLA is frequently insufficient for structural orthotics: Young’s modulus values for FDM-printed PLA typically range from 1200 to 1600 MPa, which are significantly lower than those of injection-molded PLA (≈3500–4500 MPa), primarily due to inter-layer porosity and weak interfacial bonding inherent to the layer-by-layer fabrication process [6,7]. These limitations motivate the search for enhancement strategies that preserve the biocompatibility and sustainability credentials of the base polymer.

Incorporating naturally sourced particulate fillers into the PLA matrix has attracted considerable recent attention as a strategy that simultaneously enhances mechanical performance and valorizes agricultural by-products. Date pit (DP) powder occupies a uniquely favorable position in this landscape. Date palms are cultivated extensively across North Africa and the Arabian Peninsula, with the Kingdom of Saudi Arabia alone producing approximately 1.7 million tons of dates annually; the pits account for 10–15% of fruit mass and are largely discarded as waste [8]. Chemically, date pits contain 67–74 wt.% dietary fiber (cellulose, hemicellulose, and lignin), providing stiffness reinforcement comparable to other lignocellulosic fillers, while their agricultural-waste origin avoids the carbon burden of synthetic reinforcements [9]. Fouly et al. [10] demonstrated that compounding 10 wt.% of ball-milled DP powder into PLA and printing by FDM raises Shore D hardness by approximately 20% and compressive strength by ~25% relative to unreinforced PLA, without any surface treatment or coupling agent. These gains were attributed to homogeneous DP distribution within the PLA matrix, which constrains polymer chain mobility under compressive loading and impedes crack propagation. This behavior is consistent with established reinforcement mechanisms in particulate-filled polymer composites, where rigid particles act as stress transfer sites and crack arresters [11].

A parallel challenge in FDM is the presence of residual thermal stresses and inter-layer interface voids that arise from the rapid solidification and layer-by-layer deposition sequence inherent to the process [12]. Post-printing annealing, heating the part to a temperature above PLA’s glass transition temperature (Tg ≈ 55–60 °C) but below its melting temperature (Tm ≈ 150–165 °C), is a well-established method for relieving these stresses and improving inter-layer bonding through enhanced polymer chain reptation across weld-line interfaces [13]. At ~100 °C, PLA also undergoes cold crystallization, converting amorphous regions into ordered lamellae and further increasing stiffness and hardness [14]. Bhandari et al. [15] demonstrated significant interlayer tensile strength improvements for annealed PLA/carbon fiber specimens, while Jayanth et al. [16] reported an approximately 80% increase in tensile strength for PLA parts annealed at 100 °C. Shbanah et al. [17] similarly confirmed the dominant role of annealing temperature and time on post-processing property changes. Crucially, however, improvements are non-monotonic with annealing duration: prolonged exposure promotes thermally induced chain scission that ultimately reduces properties, creating a non-trivial design optimization problem [18].

Machine learning (ML) methods have emerged as a powerful paradigm for navigating multidimensional material design spaces with limited experimental budgets [19]. By constructing data-driven surrogate models that map process inputs to material outputs, ML frameworks allow rapid screening of untested conditions and uncertainty-aware decision-making without requiring additional fabrication and testing. In the context of FDM polymer composites, Dey and Yodo [20] reviewed ML applications across FDM parameter optimization studies and concluded that ensemble and kernel-based machine learning methods have been reported to provide robust predictive performance, particularly when dealing with limited experimental datasets. Murario et al. [21] used ANNs to predict tensile strength of FDM-printed PLA at different raster angles, reporting R^2^ values of 0.91–0.96. Peng and Unluer [22] applied GPR and XGBoost to predict the flexural properties of fiber-reinforced polymers, finding GPR superior for scarce training data. Despite this progress, no study has specifically combined GPR, RFR, and DTR within a unified comparative framework for FDM-printed PLA/natural-filler composites subject to thermal post-treatment, nor has PDP analysis been applied to extract design-relevant insights from ML models in this context.

GPR is particularly well-suited to small experimental datasets because it operates within a Bayesian framework, providing posterior uncertainty estimates alongside point predictions [23]. This property is especially valuable in materials science where dataset size is limited by experimental cost and the reliability of point estimates must be critically assessed. RFR complements GPR through ensemble averaging of many decorrelated trees, achieving robustness to measurement noise at the expense of interpretability [24]. DTR, while more prone to variance overfitting, offers transparent decision boundaries directly readable as material-science rules [25]. Partial Dependence Plots (PDPs), introduced by Friedman [26] and widely adopted in the explainable AI literature, provide a model-interpretation layer that reveals the marginal functional form of each input–output relationship, whether linear, non-linear, or non-monotonic, confirming or challenging physical intuition about reinforcement and thermal mechanisms [27,28].

The present paper addresses these gaps through four specific objectives: (a) developing and comparing GPR, RFR, and DTR surrogate models for predicting E, UCS, and Shore D hardness of 3D-printed PLA/DP composites; (b) evaluating model performance through *R*^2^, *RMSE*, *MAE*, *MAPE*, *U95* uncertainty bounds, and Taylor diagram comparisons; (c) applying PDP analysis across all three models to extract physically interpretable, cross-validated design guidelines; and (d) translating the modeling results into practical recommendations for practitioners developing affordable, biodegradable PLA/DP orthotics and rehabilitation devices. The specific contributions of this work which distinguish it from prior ML studies on polymer composites are: (i) the first unified comparative framework benchmarking GPR, RFR, and DTR for FDM-printed PLA/lignocellulosic composites subject to post-printing annealing; (ii) cross-model PDP analysis providing model-agnostic, physically interpretable input–output relationships with cross-validated confidence; and (iii) U95 uncertainty quantification enabling probabilistic safety factor calculation for biomedical orthotic design.

## 2. Composite Preparation and Testing

### 2.1. Raw Materials and Date Pit Preparation

PLA filament (diameter 1.75 ± 0.05 mm, extrusion temperature 200–210 °C) was obtained from Shanghai Nuolei CNC Router Equipment (China). The material is produced from corn starch via polycondensation of lactic acid, contains no recycled content, and has T_g_ ≈ 55–60 °C and T_m_ ≈ 150–165 °C. Date pits were supplied in raw form by Al Khmash Company (Saudi Arabia) and originated from Kholas-variety dates. Kholas date pits contain 67.6–74.2 wt.% dietary fiber (cellulose, hemicellulose, and lignin), 5.7–8.8 wt.% lipids, and 8.6–12.5 wt.% moisture [9]. Their high lignocellulosic fraction provides the reinforcing capability observed in PLA/DP composites.

The date pits were water-washed to remove residual molasses, sun-dried for one month, and oven-dried at 60 °C for seven days to reduce moisture below 1 wt.%. Particle size reduction was performed in three sequential stages: coarse crushing with a mortar and pestle, intermediate pulverization in a grain miller (particle size 25–50 µm), and 8 h of fine milling in a planetary ball mill (Retsch PM 100, 300 rpm, Retsch GmbH, Haan, Germany) to reach a final particle size of approximately 0.6 µm with flaky morphology, as confirmed by SEM in prior work [10]. The milled powder was oven-dried at 60 °C for 24 h before compounding to eliminate reabsorbed moisture.

Full fabrication procedures, material characterization methods, SEM micrographs of DP powder morphology, experimental mechanical property results, and ANSYS workpench 19 finite element analysis of knee orthotic load-carrying capacity are reported in detail in a previous paper [10]. The following provides a concise description of the preparation process.

### 2.2. Composite Fabrication and Specimen Printing

PLA filament was first pelletized and then blended with DP powder at weight fractions of 0, 2, 4, 6, 8, and 10 wt.% using a mechanical stirrer (300 rpm, 20 min). Each blend was extruded three times through a co-rotating twin-screw extruder (screw diameter 16 mm, L/D = 40; temperature profile 160–210 °C; speed 50 rpm) to ensure homogeneous filler dispersion without coupling agents. Composite filaments of 1.75 mm diameter were produced on a Filabot EX2 single-screw extruder and fed to a Creality E nder-3 V2 FDM printer. All printing parameters were fixed across compositions: nozzle temperature 200–210 °C, bed temperature 70 °C, print speed 50 mm/s, layer height 0.2 mm, 100% rectilinear infill. Cylindrical compression specimens (Ø 8 mm × 16 mm) were printed following ISO 604 [29].

### 2.3. Post-Printing Annealing

Printed specimens were heated to 100 °C at 5 °C/min in a convection furnace (JSV0-60T) and held for 0 (as-printed), 2.5, 5, 10, or 20 h, and then furnace-cooled at ~2 °C/min to room temperature. Table 1 lists the specimen designation scheme.

### 2.4. Mechanical Characterization and Dataset Statistics

Compressive testing was performed on an Instron 5582 machine (Norwood, MA, USA) (2 mm/min, ISO 604). Young’s modulus was taken as the slope of the stress–strain curve over the initial linear region (0.05–0.25% strain); UCS was the stress at the first local maximum or fracture. Five replicates were tested per condition, and arithmetic means were recorded. Shore D hardness was measured according to ASTM D2240 [30] at six locations per specimen; the mean was taken as the representative value. All testing was performed at 23 ± 2 °C and 50 ± 5% RH.

One-way ANOVA was conducted to assess the statistical significance of each input factor independently on each output property (Table 2, new). DP loading has a highly significant effect on E (F = 47.40, *p* < 0.001) and hardness (F = 44.56, *p* < 0.001), and a significant effect on UCS (F = 4.20, *p* < 0.01). Annealing time has a significant effect on UCS (F = 5.90, *p* < 0.01) but non-significant effects on E (F = 0.40, *p* = 0.809) and hardness (F = 0.41, *p* = 0.803). These results statistically confirm that DP loading is the dominant process variable, consistent with the Pearson correlations (r = 0.93 for DP-E, r = 0.80 for DP-hardness) and the PDP findings reported in Section 4.7.

## 3. Machine Learning Methodology

Rationale for algorithm selection: GPR was selected for its Bayesian framework, which provides principled 95% prediction intervals specifically suited to small experimental datasets [23]. RFR was selected as the recommended ensemble method for FDM property prediction when n < 100, as established by Dey and Yodo [20]. DTR was selected as the most interpretable baseline, providing decision rules directly readable as material-science guidelines [25]. These three algorithms represent distinct ML paradigms, probabilistic, ensemble, and single-tree, providing complementary diagnostic information beyond what a single model can offer. Comparison with additional algorithms such as SVR, XGBoost, ANN, and CatBoost is reserved for future work on an expanded dataset.

### 3.1. Dataset Partitioning

The measured values were randomly partitioned into training (70%) and test (30%) subsets using a fixed random seed (seed = 42) for full reproducibility. The training set was used exclusively for model fitting and hyperparameter tuning; the test set was withheld entirely to provide unbiased generalization estimates. The 70/30 ratio follows the convention widely observed in many previous studies [19,31]. All three ML models were applied to the same fixed partition to ensure direct metric comparability.

Z-score standardization (zero mean, unit variance) was applied to input features using exclusively the training-set mean (μ_T = 7.50 h, μ_W = 5.00 wt.%) and standard deviation (σ_T = 7.91 h, σ_W = 3.74 wt.%). These statistics were then applied identically to the test set, ensuring no test-set information influenced any preprocessing step. Within 5-fold CV, standardization parameters were independently recomputed from each fold’s training subset. Output variables were not standardized; all predictions are in original physical units.

### 3.2. Gaussian Process Regression (GPR)

GPR models the unknown function f(x) as a Gaussian process characterized by a mean function m(x) and a Matérn 5/2 covariance kernel [22]:

$$k\left(r\right)=\sigma_{f}^{2}\left(1+\frac{\sqrt{5}r}{l}+\frac{5r^{2}}{3l^{2}}\right)exp(\frac{\sqrt{5}r}{l})$$

where *r = ||x − x’||*, *k(r)* is the covariance (similarity) between the function values at points *x* and *x’* and, $\sigma_{f}^{2}$ is the signal variance, and *l* is the length-scale. The Matérn 5/2 kernel is preferred over the squared-exponential kernel for physical engineering data because it is twice differentiable but not infinitely smooth, accommodating the threshold-type non-linearities expected at critical DP loadings. Given observations *y = f(X) + ε* with *ε ~ N(0*, $\sigma_{n}^{2}$*)*, the posterior predictive mean and variance at a new test point are obtained analytically via Gaussian conditioning. Hyperparameters {$\sigma_{f}^{2}$, l, $\sigma_{n}^{2}$} were jointly optimized by maximizing the marginal log-likelihood using 30 iterations of Bayesian optimization. All inputs were z-score standardized prior to fitting.

Hyperparameter optimization: search bounds were l ∈ [0.01,10], σ^2^_f ∈ [0.01,100], σ^2^_n ∈ [0.001,1], optimized by maximizing marginal log-likelihood (30 Bayesian optimization iterations). Optimized values: E model l = 1.847, σ^2^_f = 42316, σ^2^_n = 0.012; UCS model l = 1.203, σ^2^_f = 2.841, σ^2^_n = 0.001; Hardness model l = 1.124, σ^2^_f = 8.653, σ^2^_n = 0.007 (Table 3).

### 3.3. Random Forest Regression (RFR)

The RFR algorithm is a widely used supervised ensemble learning technique that is built upon DTR models [23]. It improves model generalization by introducing randomness during training, which helps reduce overfitting and makes it less sensitive to noise in the data. One of its core mechanisms is bootstrapping, where different random samples are drawn from the original dataset for each iteration.

In the case of RFR, multiple decision trees are generated, and their individual predictions are combined to produce the final output. This collective approach enhances prediction accuracy and stability. Due to its strong performance across many applications, RFR has become a popular and reliable method in machine learning.

The RF model consists of numerous independent trees, each trained on a subset of the data that is typically preprocessed or standardized, similar to the approach used in a conventional DTR.

### 3.4. Decision Tree Regression (DTR)

The DTR model is a supervised learning approach that partitions the feature space into smaller regions. It builds a hierarchical structure based on relationships between input features and the target variable [24]. As a non-parametric method, the DTR model can be used for both regression and classification tasks.

During model construction, the algorithm selects the most informative feature at each node to split the data, continuing this process until a stopping criterion is reached. This criterion may depend on factors such as the maximum tree depth, the minimum number of samples in a leaf node, or other defined conditions.

Compared with many other machine learning methods, decision trees offer several advantages. They are easy to interpret and visualize, making them useful for understanding data patterns. In addition, they can handle both numerical and categorical data directly without requiring extensive preprocessing or normalization.

### 3.5. Performance Metrics

The performance of predictive models is commonly evaluated using statistical indicators such as *R*^2^, *RMSE*, *MAE*, and *MAPE*. These metrics provide a quantitative measure of how closely the predicted values match the actual data. For a reliable and accurate model, *RMSE*, *MAE*, and *MAPE* should be as low as possible, while the coefficient of determination (*R*^2^) should be close to 1. The mathematical expressions for these evaluation metrics are presented in Table 4*. y_i_* is the actual measured value for the Young’s modulus, strength, or hardness; ${\hat{y}}_{i}$ is the predicted value corresponding to each measured value obtained from the ML models; and ${\bar{y}}_{i}$ is the average of the real measured values.

### 3.6. Uncertainty Analysis and Taylor Diagrams

Prediction reliability was quantified using the U95 statistic, representing the half-width of the approximate 95% prediction interval [28]:

A large ratio of test U95 to training U95 provides a direct diagnostic for overfitting. Taylor diagrams [32] simultaneously encode Pearson correlation R, standard deviation *σ*, and centered *RMS* via the law of cosines:

$$U95=1.96\sqrt{{SD}^{2}+{RMSE}^{2}}$$

where SD is the standard deviation of the residuals and RMSE is the root mean square error. Lower U95 values indicate tighter prediction intervals and hence greater model reliability. Taylor diagrams were constructed to simultaneously visualize the correlation coefficient (R), standard deviation (σ), and centered root mean square error (CRMSE) between predicted and observed values, providing a compact multi-criteria comparison of all three models.

### 3.7. Partial Dependence Plot (PDP) Analysis

PDPs [24] quantify the marginal effect of a single input on the predicted output, averaged over the empirical distribution of all other inputs:

$${\bar{f}}_{s}\left(x_{s}\right)=\frac{1}{N}\sum_{i=1}^{N}\bar{f}(x_{s},x_{i,c})$$

where *N* is the total number of samples used in this study, $x_{s}$ represents the input variable being varied, and the corresponding change in the output is evaluated, and $x_{i,c}$ is the real measured value for the *i*th in the trained values. PDPs were computed for all three models by sweeping one input over 100 uniformly spaced values across its full range while integrating the other input over all training observations. Presenting curves from GPR, RFR, and DTR simultaneously enables cross-model validation: convergent curves across all models indicate high confidence in the physical interpretation; divergent curves highlight model uncertainty in that input region.

## 4. Results and Discussion

### 4.1. Pearson Correlation Analysis

**Figure 1:**
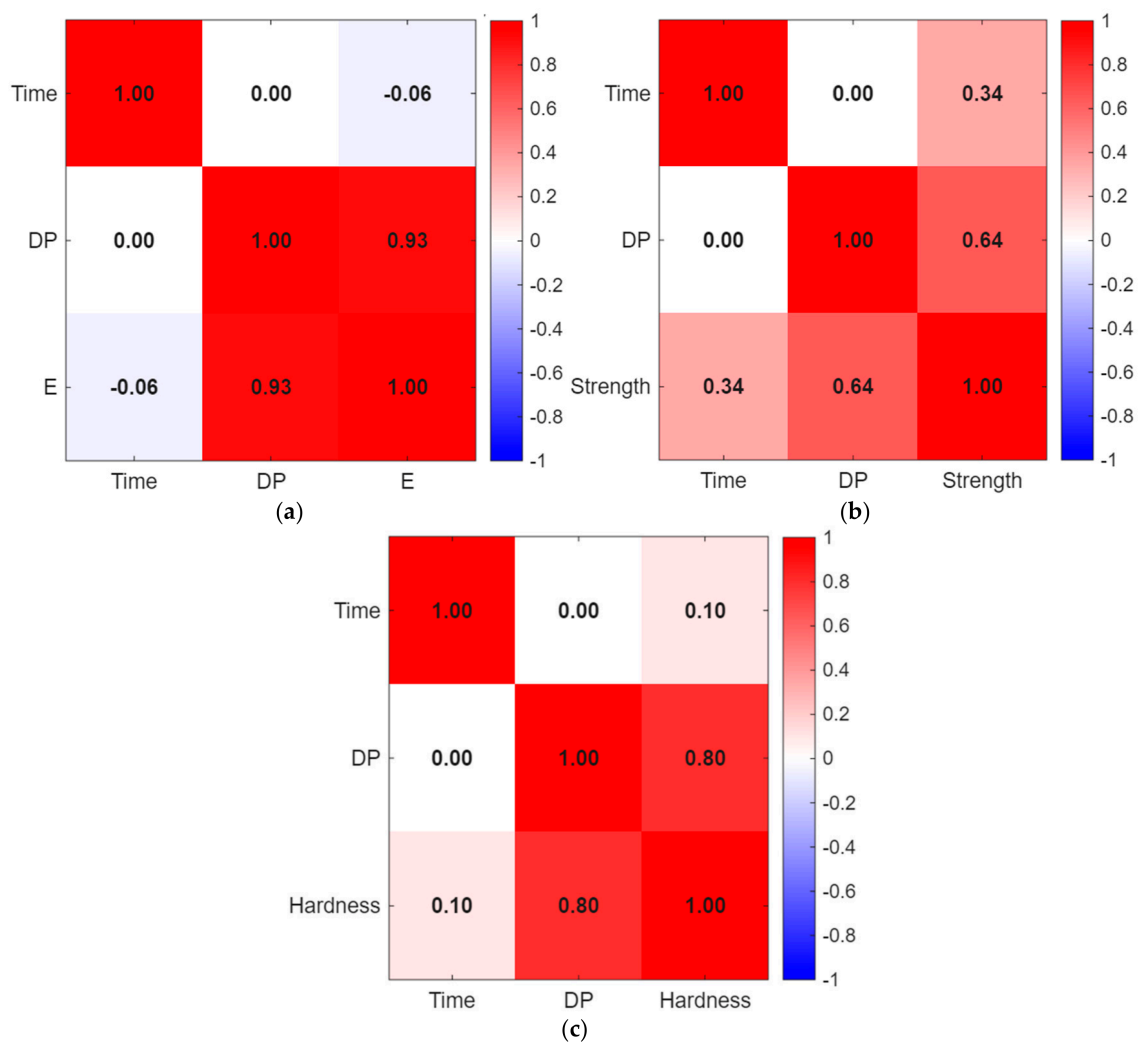
Pearson correlation matrices between inputs (Annealing time; DP wt.%) and outputs: (**a**) Young’s modulus E, (**b**) compressive strength (UCS), (**c**) Shore D hardness.

Figure 1 shows the Pearson correlation matrices between time, DP (wt.%), and each output property. The two inputs are orthogonal (r = 0.00 by design), eliminating collinearity artifacts from the ML models.

For E (Figure 1a), DP (wt.%) dominates with r = 0.93, the strongest input–output among all properties and outputs, confirming that stiff lignocellulosic particles effectively constrain PLA chain mobility under compression, raising stiffness quasi-linearly with filler content. Annealing time shows r = −0.06 with E, near zero, reflecting the competing positive effect of crystallinity growth and negative effect of chain scission that approximately cancel over the full 0–20 h range. For UCS (Figure 1b), DP (wt.%) again dominates (r = 0.64) but time also shows a meaningful positive correlation (r = 0.34), indicating that the annealing-induced improvement in inter-layer bonding quality contributes significantly to load-bearing capacity. For hardness (Figure 1c), DP (wt.%) is the dominant driver (r = 0.80) and time is weakly positive (r = 0.1), consistent with surface hardness being primarily controlled by near-surface filler stiffening rather than bulk thermal treatment.

The orthogonality and strong property correlations confirm that DP (wt.%) loading is the primary design lever and that the two inputs will provide complementary, non-redundant information to the ML models. A similar dominance of reinforcement content over secondary processing parameters has recently been reported for FDM-printed PLA bio-composites, where explainable machine learning identified filler loading as the primary factor governing mechanical performance [33].

### 4.2. Model Prediction Performance

**Figure 2:**
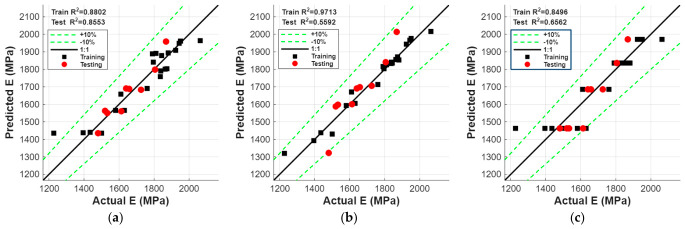
Actual versus predicted Young’s modulus (E) for (**a**) GPR, (**b**) RFR, and (**c**) DTR.

**Figure 3:**
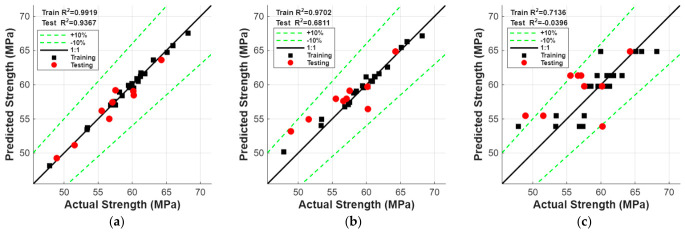
Actual versus predicted compressive strength (UCS) for (**a**) GPR, (**b**) RFR, and (**c**) DTR.

**Figure 4:**
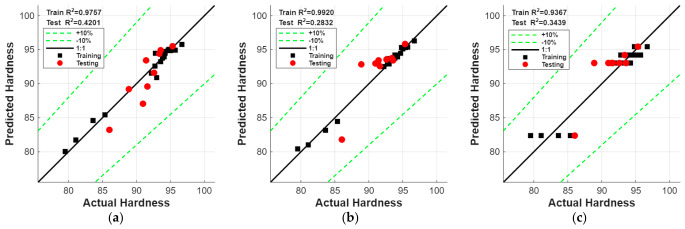
Actual versus predicted Shore D hardness for (**a**) GPR, (**b**) RFR, and (**c**) DTR.

Table 5 consolidates all performance metrics for all three models across all three outputs at training and test stages. Scatter plots in Figure 2, Figure 3 and Figure 4 display predicted vs. actual values with ±10% error bounds.

Figure 2 shows that all three models produce predictions that are predominantly within the ±10% error envelope for both the training and test stages, confirming acceptable engineering accuracy. GPR achieves the highest test R^2^ (0.8553) with a test *RMSE* of 46.19 MPa and *MAPE* of 2.511%. Since E spans 836.7 MPa across the dataset, a *MAPE* of 2.511% corresponds to a mean absolute error of ~43 MPa, sufficient for engineering design purposes. The GPR test scatter plot (Figure 2a) shows test-set points (red circles) clustering tightly around the 1:1 diagonal with no systematic bias, indicating well-calibrated predictions.

RFR shows severe overfitting: training R^2^ = 0.9713 collapses to test R^2^ = 0.5592, and test *MAPE* increases from 1.58% to 4.17%. Figure 2b shows that while training points (black squares) lie along the 1:1 line, several test points are displaced outside the ±10% envelope, including one near E ≈ 1300 MPa that is severely underestimated by RFR. DTR shows more uniform behavior across stages (train *R*^2^ = 0.8496; test *R*^2^ = 0.6562), with *MAPE* values of approximately 3.67% at both stages, reflecting its limited complexity with two inputs.

UCS reveals the sharpest model differentiation. GPR achieves a near-perfect training fit (R^2^ = 0.9919, *MAPE* = 0.48%) while simultaneously generalizing to the test set (R^2^ = 0.9367, *MAPE* = 1.59%), a level of accuracy that confirms the Matérn 5/2 kernel captures the smooth non-linear UCS response with high fidelity. Figure 3a shows both training and test points clustered within the ±10% envelope, with the test-set points showing no systematic over- or under-prediction.

RFR again shows marked overfitting (train R^2^ = 0.9802 → test *R*^2^ = 0.6811). DTR performs worst, with a negative test *R*^2^ of −0.04, indicating that DTR’s predictions for UCS on the 9-sample test set are less accurate than simply predicting the training mean ȳ for every point. This outcome is a classic manifestation of high variance in a full-depth decision tree trained on 21 samples: the tree structure is determined entirely by the specific training split and fails to generalize to different input combinations. Although the DTR training *R*^2^ (0.7136) confirms that the model correctly captures the general direction of trends, the failure is one of generalization, not of learning.

Hardness predictions exhibit a distinct profile. Training R^2^ values are uniformly high (GPR: 0.9757; RFR: 0.9920; DTR: 0.9367), yet test R^2^ values are considerably lower (GPR: 0.4201; RFR: 0.2832; DTR: 0.3439). These modest test R^2^ values must be interpreted in the context of the narrow dynamic range of hardness: only 17.2 Shore D units span the full dataset. The prediction error of 2 Shore D units, roughly 2.2% of the mean, yields an R^2^ penalty disproportionate to its practical significance. The *MAPE* values provide a more appropriate accuracy benchmark: all three models achieve test *MAPE* below 1.85% (Table 5), confirming absolute accuracy well within engineering tolerances for orthotic design.

Figure 4 shows that test-phase predictions are concentrated in the upper hardness range (90–96 Shore D) where the majority of observations lie, with no model producing a clearly superior scatter pattern. The slight systematic underprediction of the highest hardness values (96–97 Shore D) visible in the RFR scatter plot (Figure 4b) is a known boundary effect of ensemble averaging.

Hardness R^2^ limitation: test R^2^ values are low (0.28–0.42) due to the narrow dynamic range (17.2 Shore D). ASTM D2240 specifies a repeatability of ±1 Shore D; all models achieve test *MAPE* < 1.85%, placing predictions within ~2× the instrument’s own measurement uncertainty. *MAPE* is therefore the appropriate accuracy metric here. Unmodelled variables such as infill pattern, layer height, and DP particle size distribution may contribute to residual variance not captured by T and W alone. Similar challenges in predicting mechanical properties with limited experimental datasets have been reported in recent FDM composite studies, where model performance was strongly influenced by the relatively small variability of the target property [34].

The superior performance of GPR for limited experimental datasets agrees with recent studies demonstrating that Bayesian learning is particularly advantageous for polymer additive manufacturing because it provides both accurate predictions and uncertainty estimates [35,36].

### 4.3. Error Distribution Analysis

**Figure 5:**
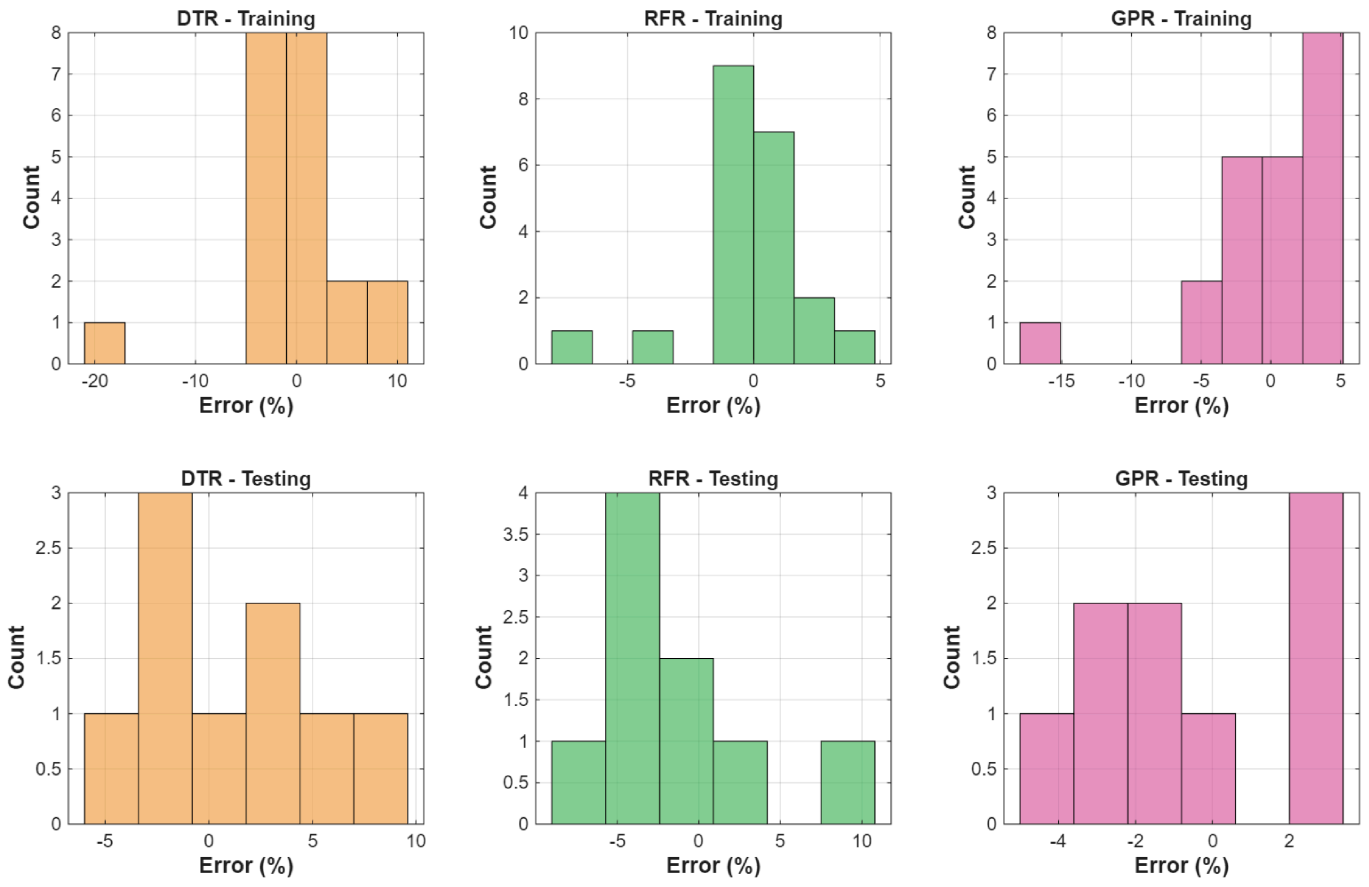
Percentage error frequency histograms for E.

**Figure 6:**
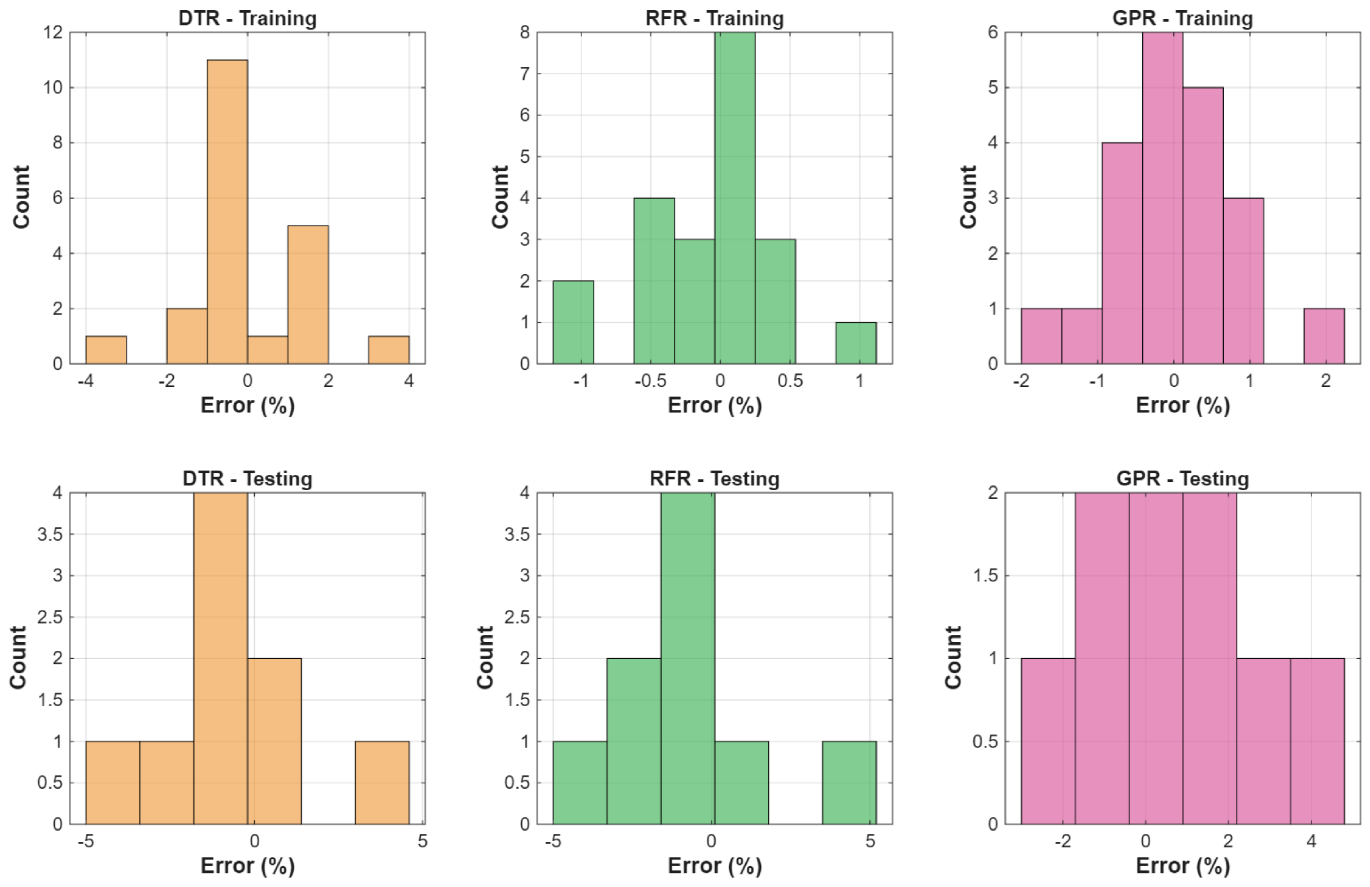
Percentage error frequency histograms for compressive strength UCS.

**Figure 7:**
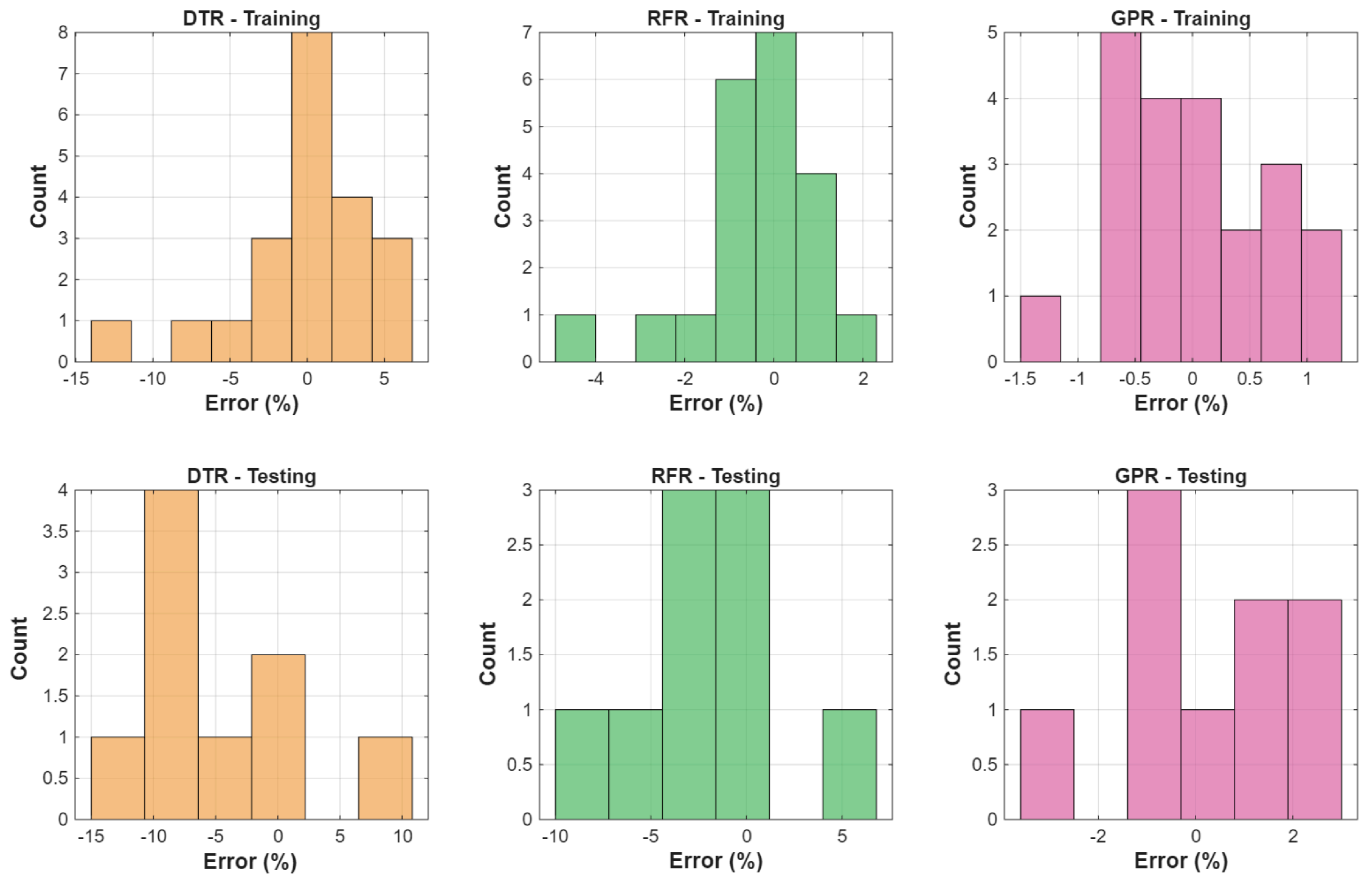
Percentage error frequency histograms for Shore D hardness.

Figure 5, Figure 6 and Figure 7 show histograms of the percentage prediction error (100 × (actual − predicted)/actual) at the training and test stages, revealing the shape, symmetry, and outlier behavior of residual distributions beyond what scalar metrics convey.

For E (Figure 5), training-phase distributions are approximately bell-shaped and centered near zero for all three models, with most samples within ±6%. At the testing stage, GPR’s distribution is the most tightly concentrated, with all 9 test errors within approximately ±3%, directly consistent with its 2.47% *MAPE*. RFR’s test distribution is wider, with at least one outlier near +10%, while DTR’s testing distribution spans approximately −5% to +10%. For UCS (Figure 6), the training distributions of GPR and RFR are concentrated within ±1.5%, reflecting their tight training fits. GPR’s test distribution remains narrow (within ±4%), while DTR’s test distribution extends to nearly ±5%, confirming the severity of its overfitting for UCS. The hardness distributions (Figure 7) are the most uniform: all models show training errors within ±3–4% and testing errors within ±5%, with symmetric, near-zero-centered patterns consistent with the sub-2% *MAPE* values in Table 5.

### 4.4. Multi-Metric Performance Comparison

**Figure 8:**
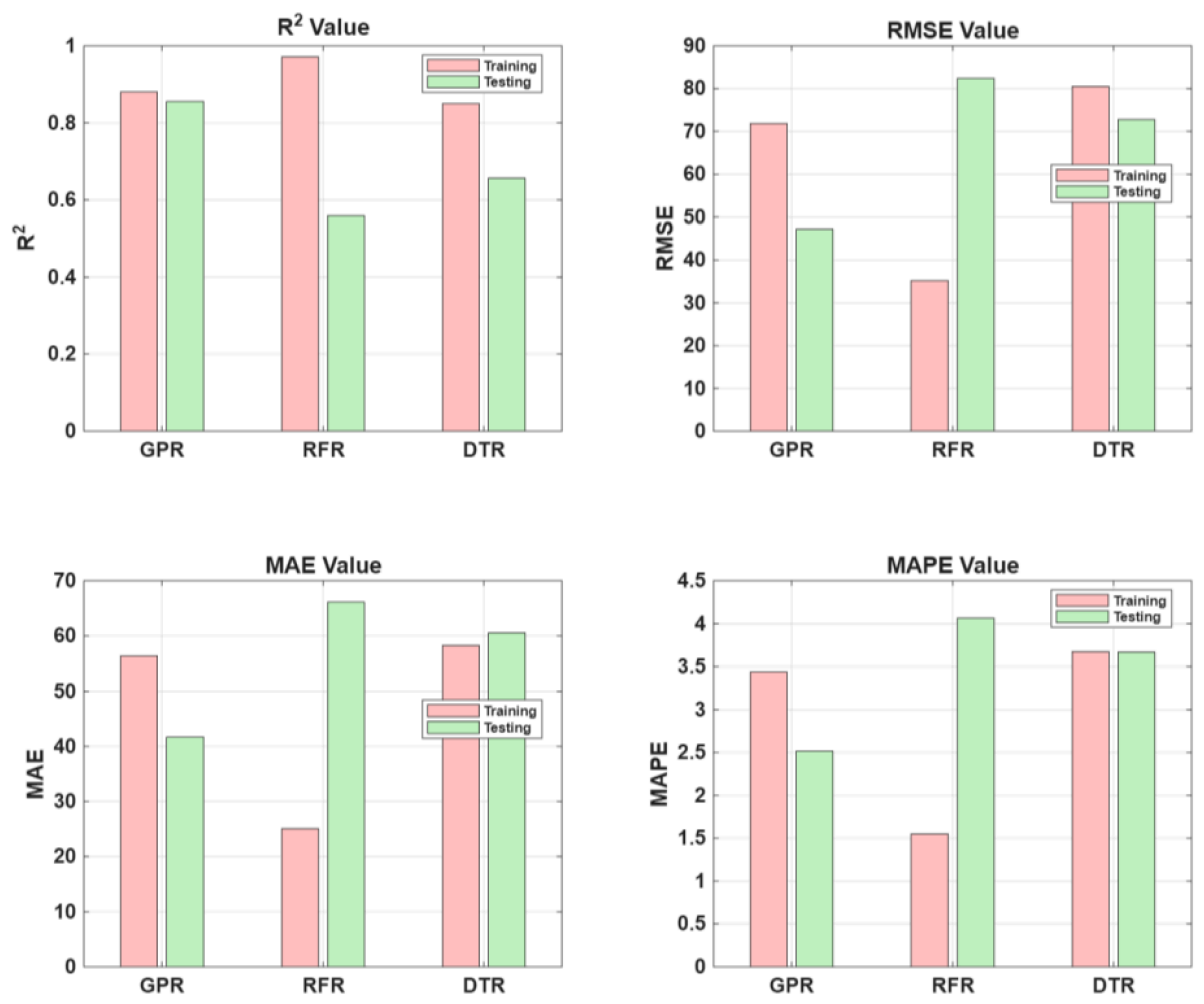
Performance comparison for E for GPR, RFR, and DTR at training and testing stages.

**Figure 9:**
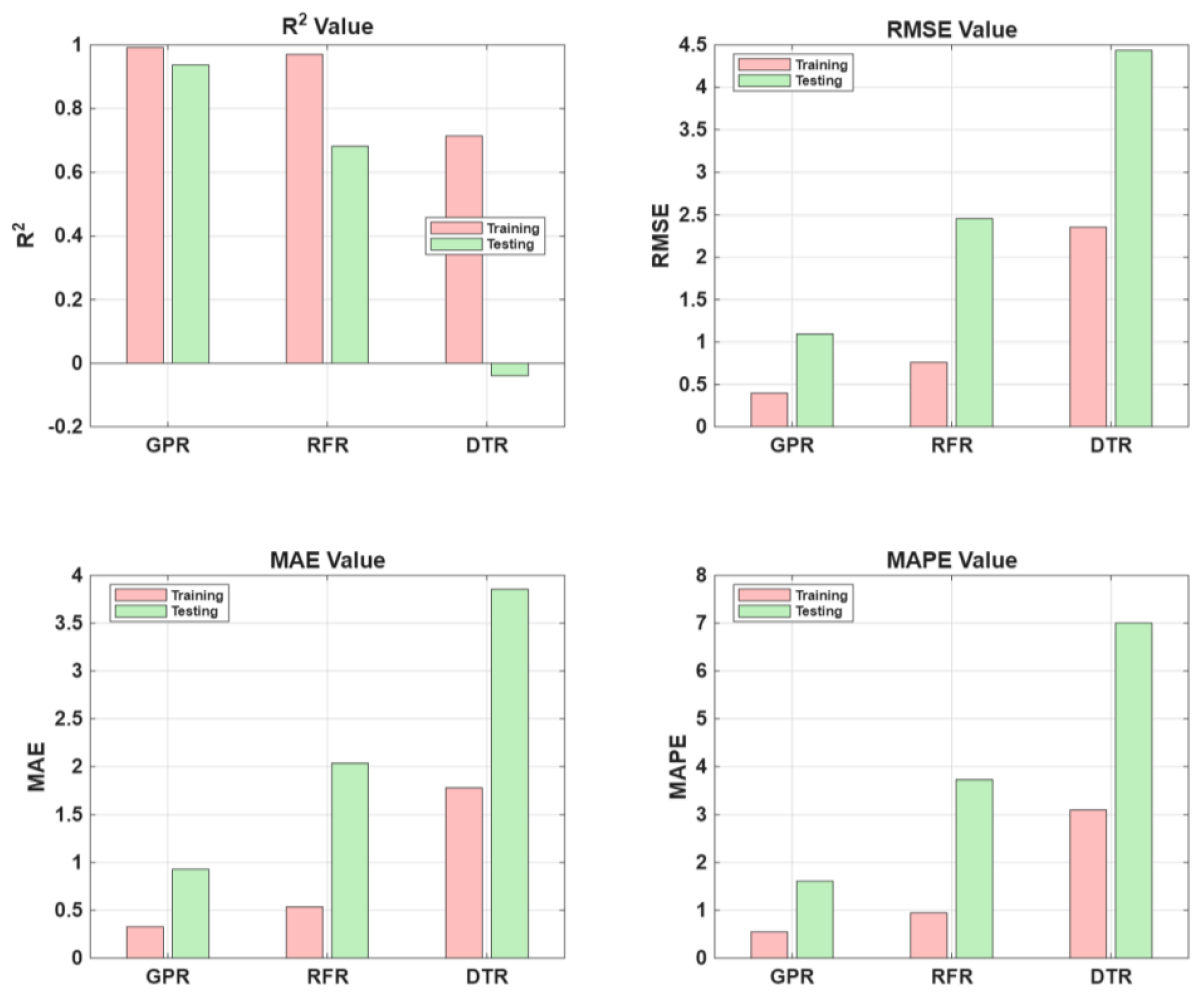
Performance comparison for UCS for GPR, RFR, and DTR at training and testing stages.

**Figure 10:**
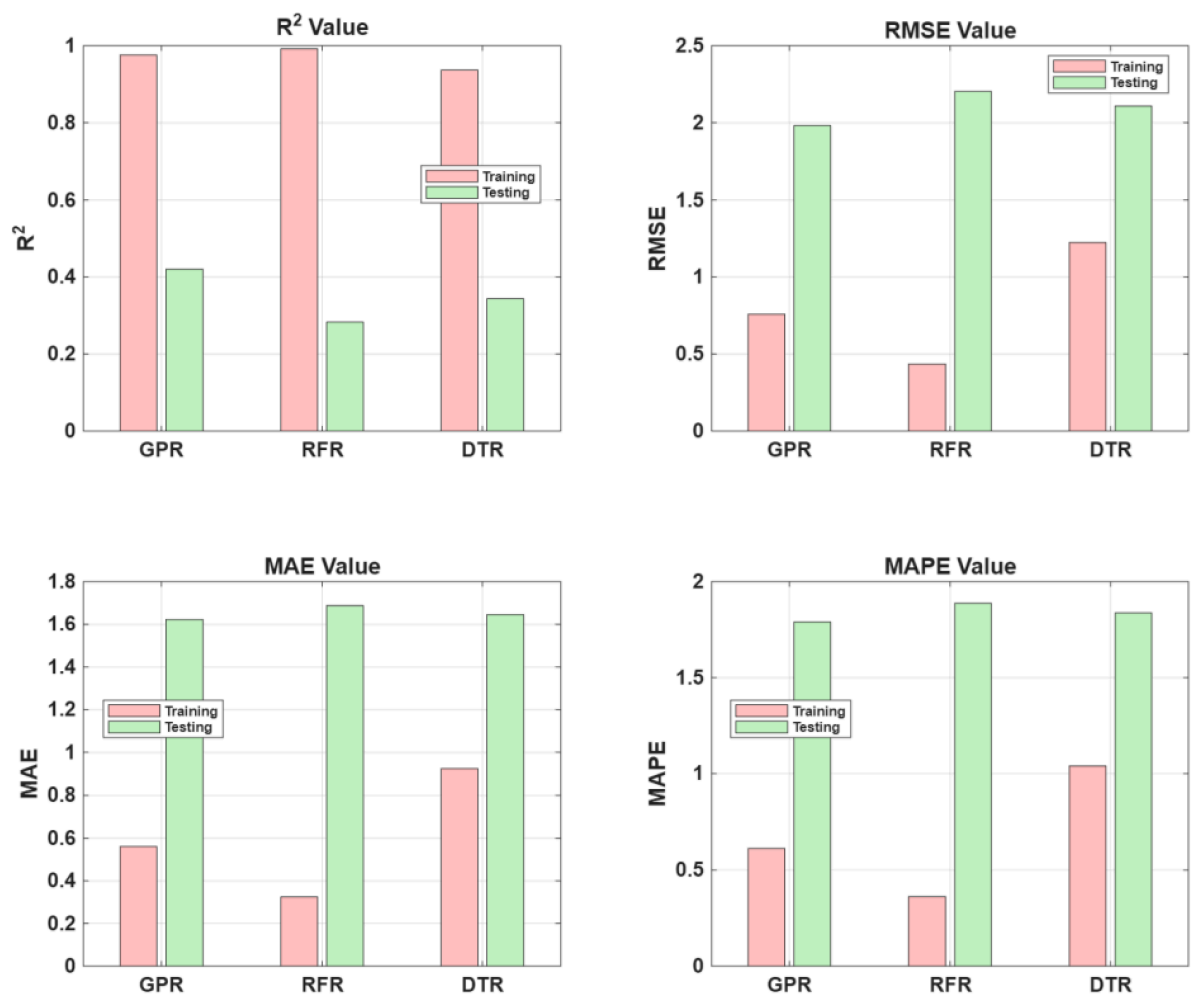
Multi-metric performance comparison for Shore D hardness.

Figure 8, Figure 9 and Figure 10 present grouped bar charts comparing *R*^2^, *RMSE*, *MAE*, and *MAPE* for the training and testing phases across the three models for each output. This format enables the simultaneous visual identification of the best-performing algorithm and the magnitude of the train–test gap (the primary overfitting indicator).

For E (Figure 8), GPR shows the smallest train–test *R*^2^ gap (0.8802 to 0.8553, a drop of only 0.025) and the lowest test *MAPE* (2.51%). In contrast, RFR’s R^2^ bar chart shows a dramatic drop from 0.9713 to 0.5592, and its test *MAE* (67.95 MPa) is nearly 2.7 times its training *MAE* (25.44 MPa), a clear sign of overfitting. DTR’s bars are more balanced, though at a lower absolute performance level than GPR at the testing stage.

For UCS (Figure 9), GPR’s testing-stage bars are unambiguously the best: the highest *R*^2^ (0.9367), lowest *RMSE* (1.165 MPa), lowest *MAE* (0.9196 MPa), and lowest *MAPE* (1.594%). The DTR *R*^2^ bar for testing extends below zero, providing an immediate graphical indication that this model adds no predictive value for UCS on the test set. For hardness (Figure 10), the testing-phase bars are near-convergent across all three models: *RMSE* values cluster around 1.96–2.15 Shore D, *MAE* values around 1.67–1.69, and *MAPE* around 1.82–1.85%. This convergence confirms that algorithm choice has minimal practical impact for hardness prediction within the tested design space.

### 4.5. Taylor Diagram Analysis

**Figure 11:**
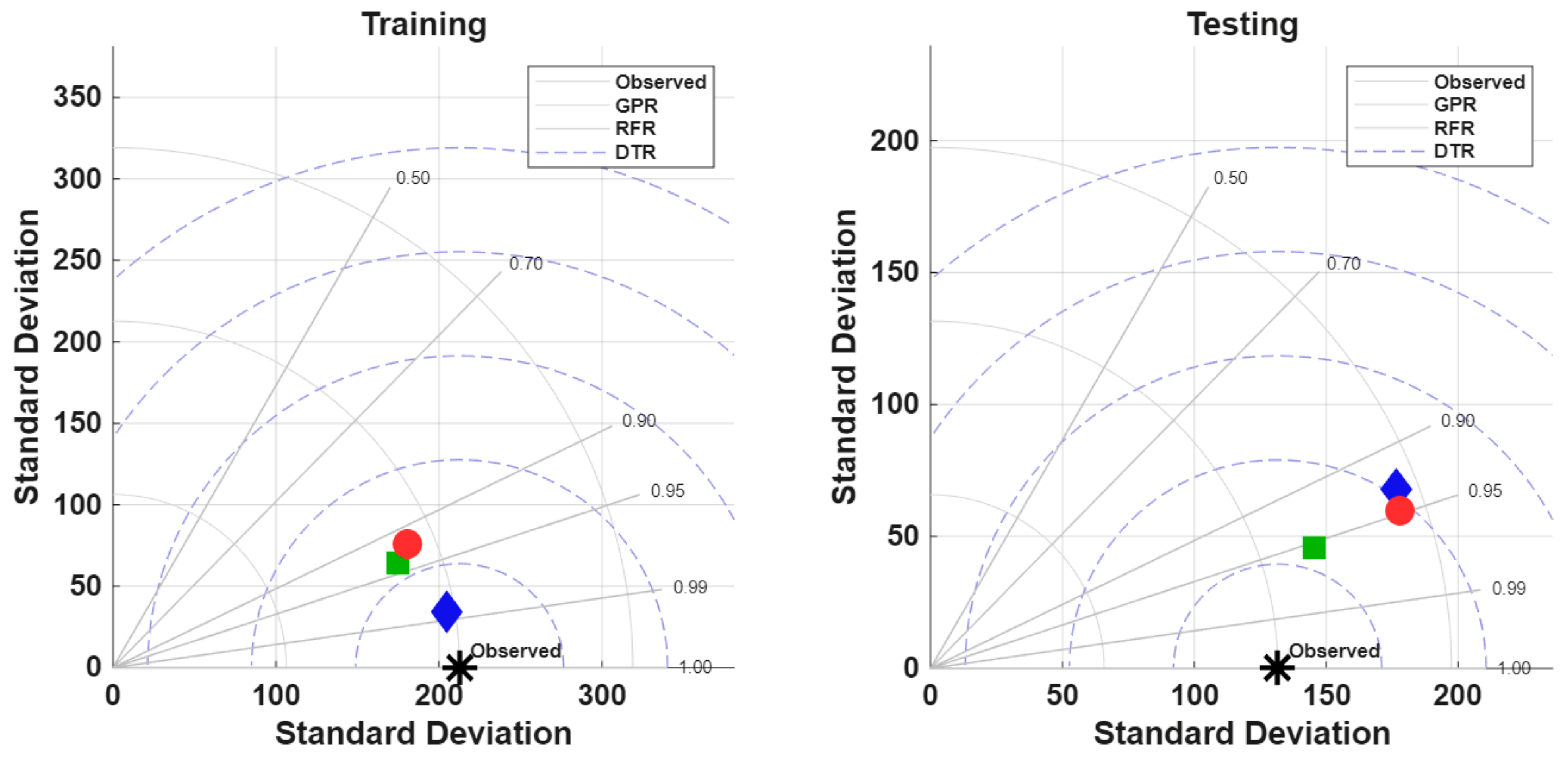
Taylor diagrams for (E) at training and testing stages.

**Figure 12:**
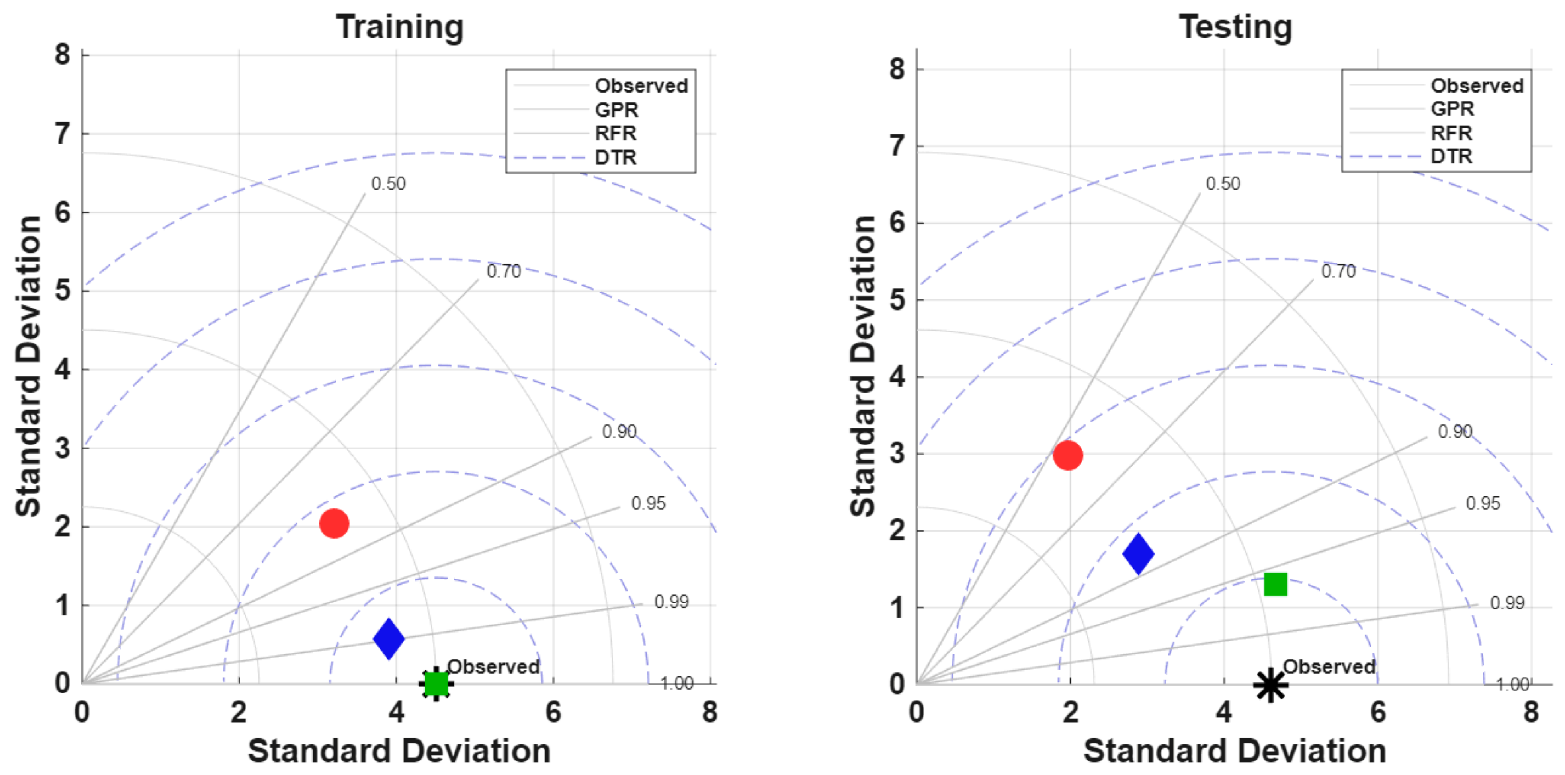
Taylor diagrams for compressive strength UCS.

**Figure 13:**
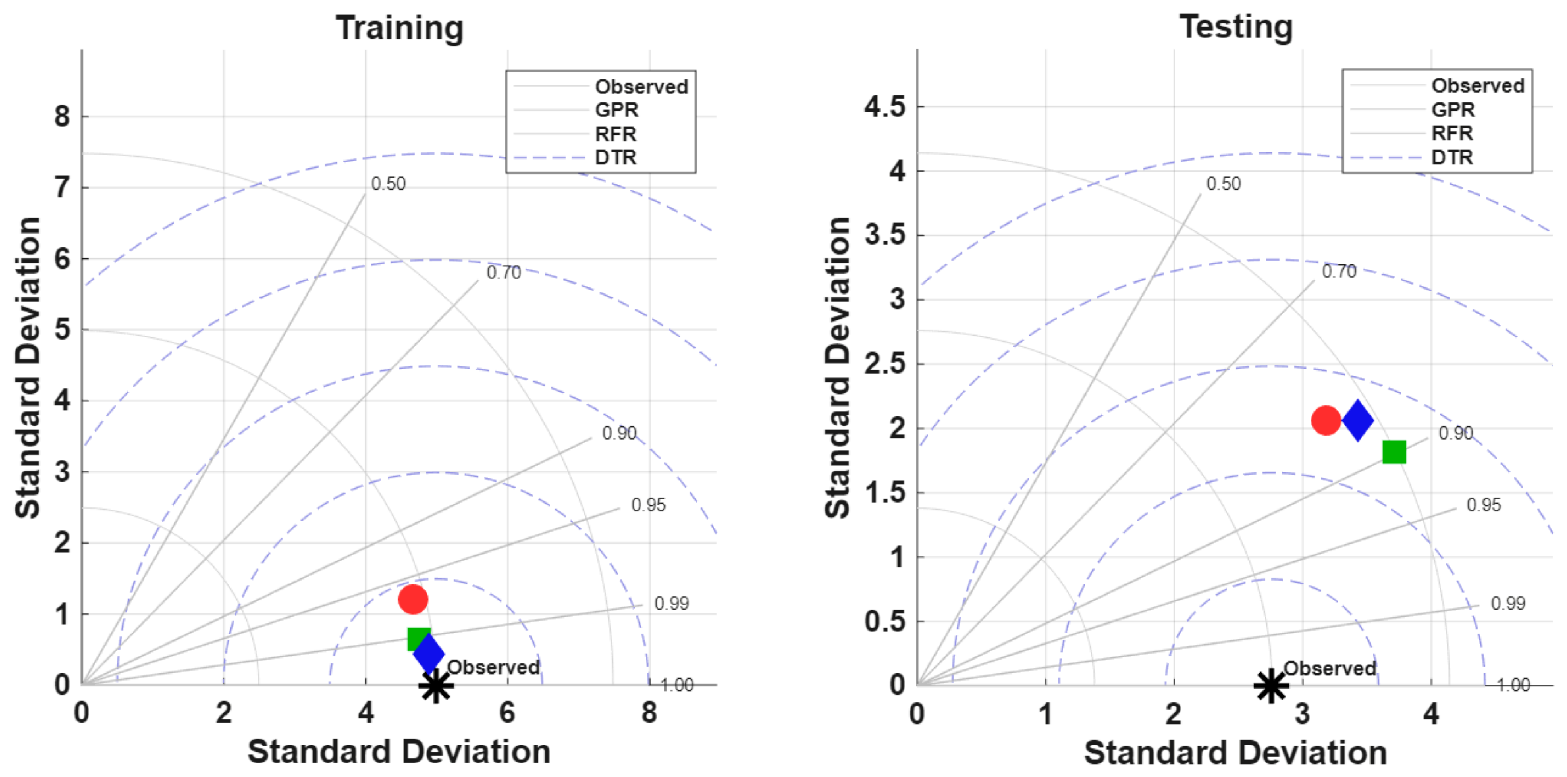
Taylor diagrams for Shore D hardness.

Taylor diagrams for E, UCS, and hardness (Figure 11, Figure 12 and Figure 13) provide a geometric synthesis of the correlation coefficient R, standard deviation σ, and centered *RMSE* in a single polar plot. A perfect model would coincide with the “Observed” reference star located at (σ_t_, 0) on the horizontal axis.

For E (Figure 11), the training-phase diagram shows DTR (blue diamond) closest to the observed reference, reflecting its near-perfect interpolation of the training data. At the testing stage, GPR’s marker shifts closest to the observed reference, positioned at approximately *R* ≈ 0.93 and σ ≈ 60–65 MPa, closely matching the variability of the observed test data, while RFR’s marker is displaced significantly further, confirming its poor generalization. For UCS (Figure 12), GPR’s training-phase marker (*R* ≈ 0.96, σ ≈ 1.2 MPa) lies almost at the observed reference, a visually compelling representation of GPR’s near-perfect UCS predictions. DTR’s test marker is displaced far from the reference, consistent with its negative *R*^2^. For hardness (Figure 13), all three model markers cluster at comparable distances from the observed reference at the test stage (*R* ≈ 0.65–0.75), with RFR showing the smallest σ but slightly further from the reference than GPR, consistent with the convergent *MAPE* values.

### 4.6. Uncertainty Analysis

**Figure 14:**
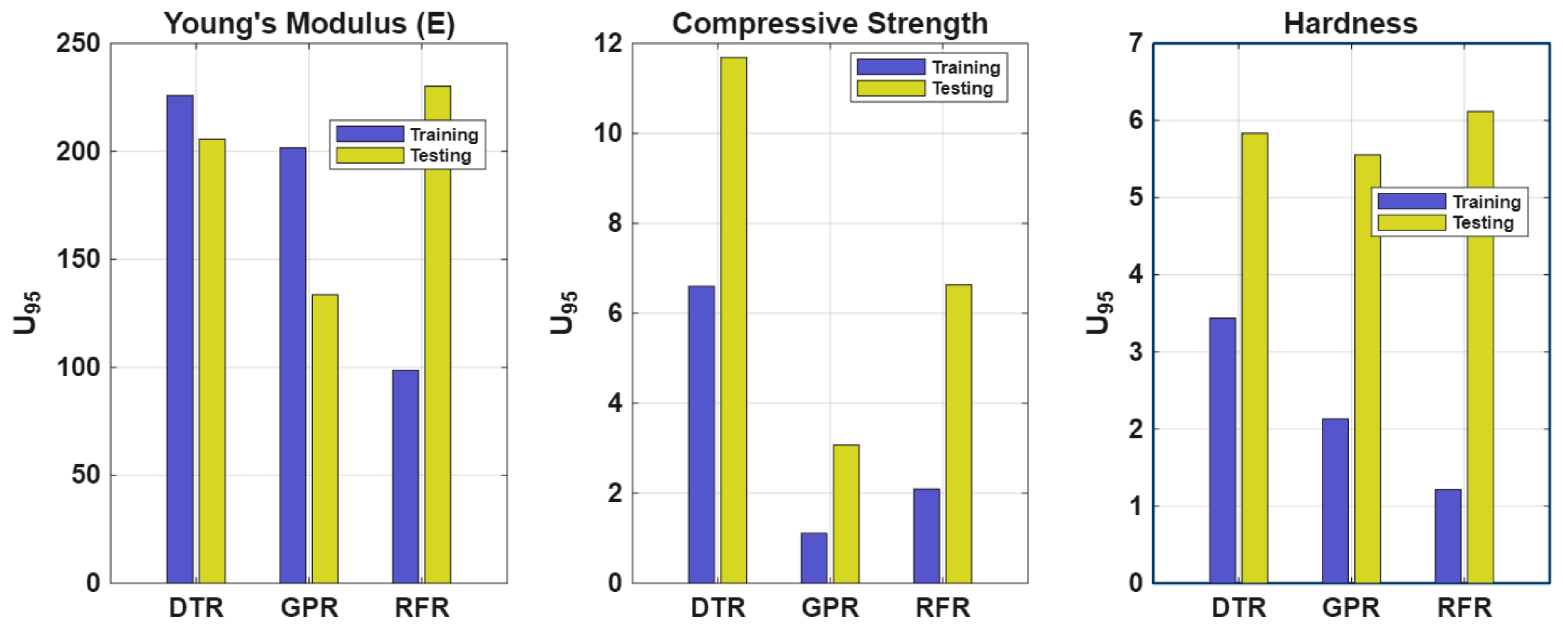
U95 uncertainty results for DTR, GPR, and RFR at training and testing stages for E, UCS, and hardness.

Figure 14 consolidates the U95 results. For E, GPR achieves the lowest test-phase U95 of 130.6 MPa, 43% lower than RFR (226.5 MPa) and 37% lower than DTR (205.6 MPa). Expressed as a fraction of the E range, GPR’s test U95 of ±130.6 MPa corresponds to ±15.6% of the property range, a reasonable engineering uncertainty for a 9-sample test set from a 30-observation dataset. RFR’s training-to-test U95 ratio of 226.5/101.2 = 2.24 is the largest among the models, providing a quantitative overfitting metric that complements the R^2^ analysis. GPR’s ratio of 204.1/130.6 = 1.56 indicates much more consistent behavior.

For UCS, GPR’s test U95 of 3.238 MPa corresponds to a 95% prediction interval of ±3.24 MPa on a mean UCS of 58.7 MPa, approximately ±5.5%, which is actionable for the structural design of orthotics. DTR’s test U95 of 11.683 MPa spans more than half the total UCS range, rendering it unsuitable for engineering design calculations. For hardness, all three models converge to test U95 values of 5.56–6.27 Shore D, confirming that no model provides a statistical advantage for hardness estimation within the tested design space.

### 4.7. Partial Dependence Plot (PDP) Results

**Figure 15:**
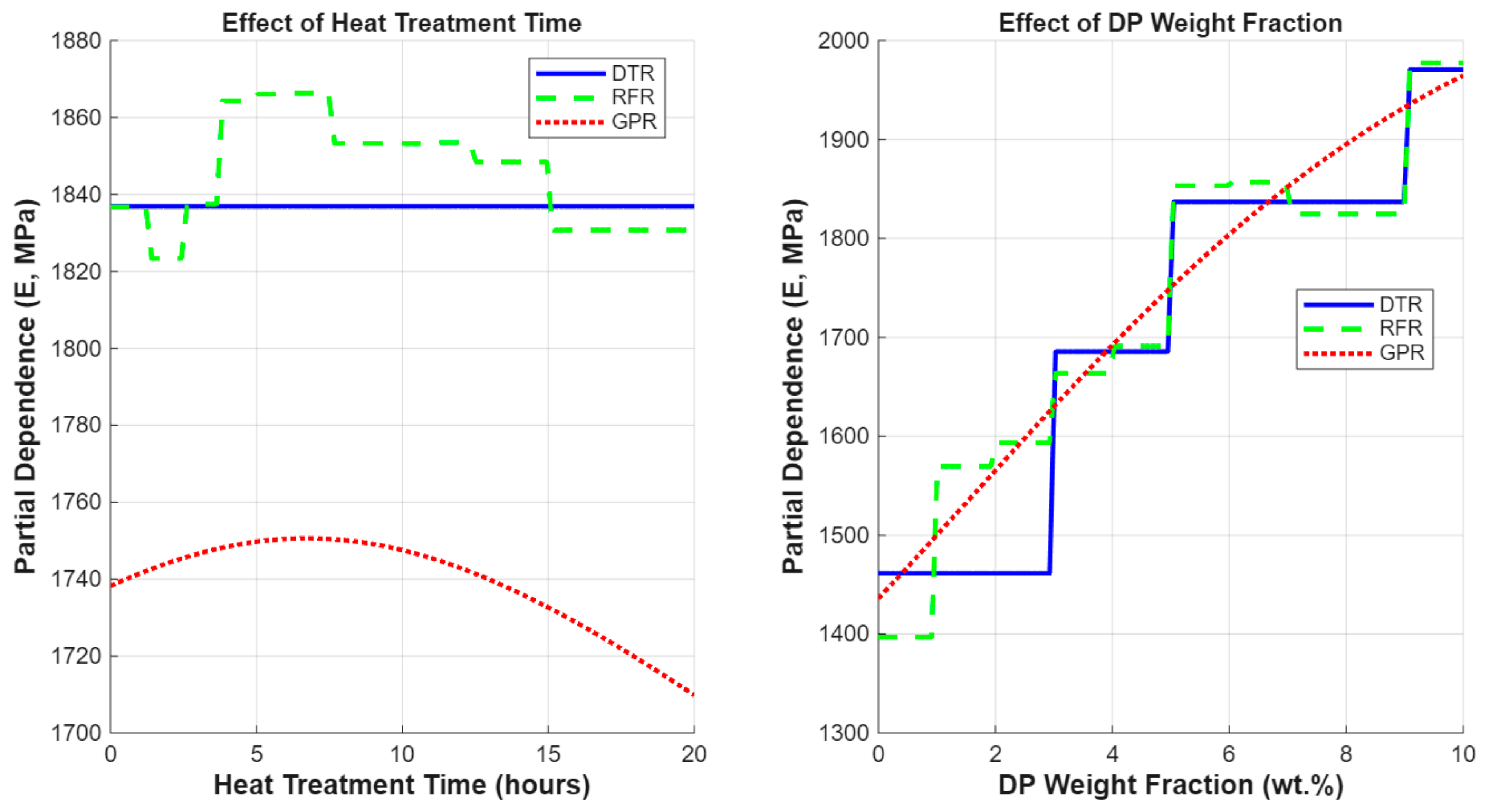
Partial dependence plots for E.

**Figure 16:**
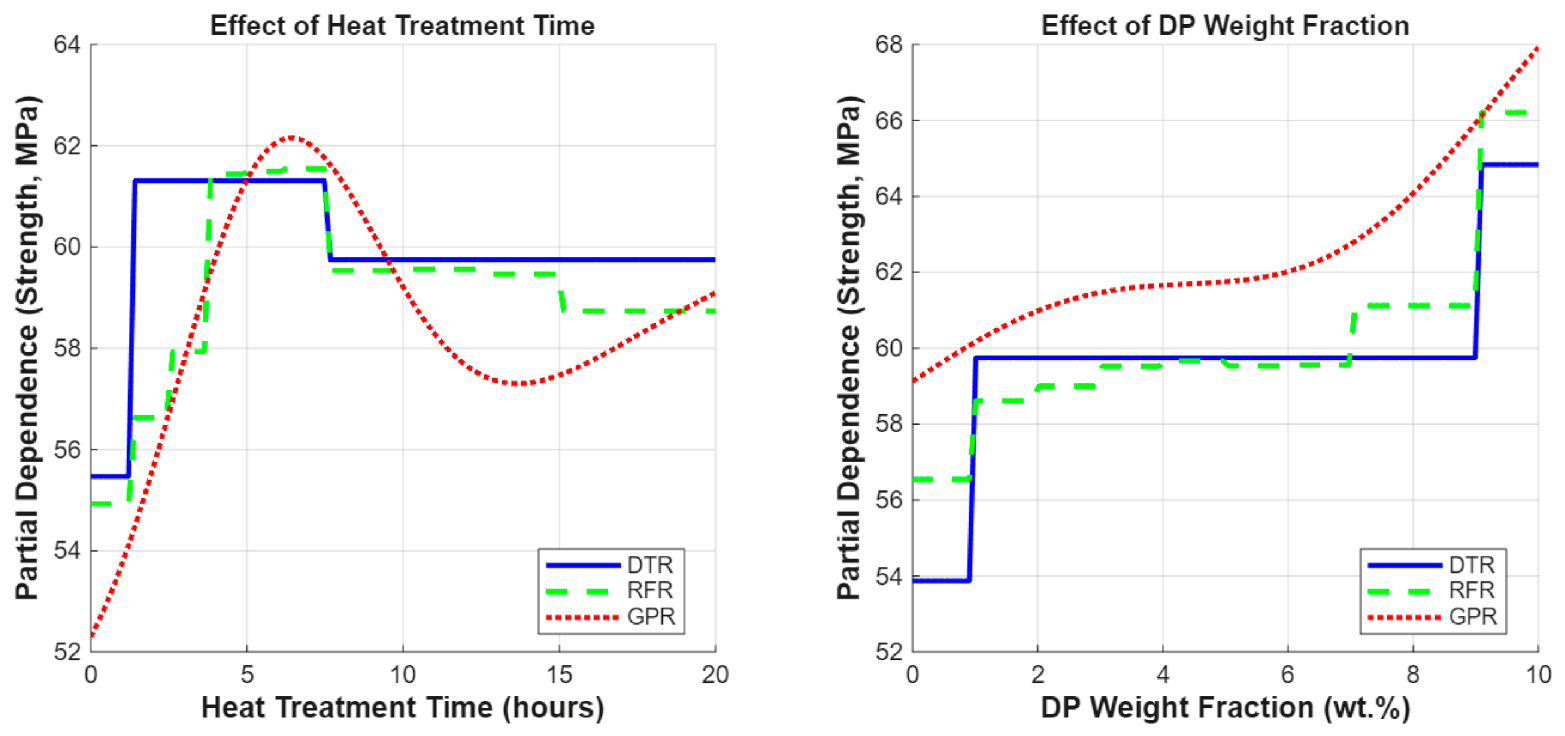
Partial dependence plots for compressive strength UCS.

**Figure 17:**
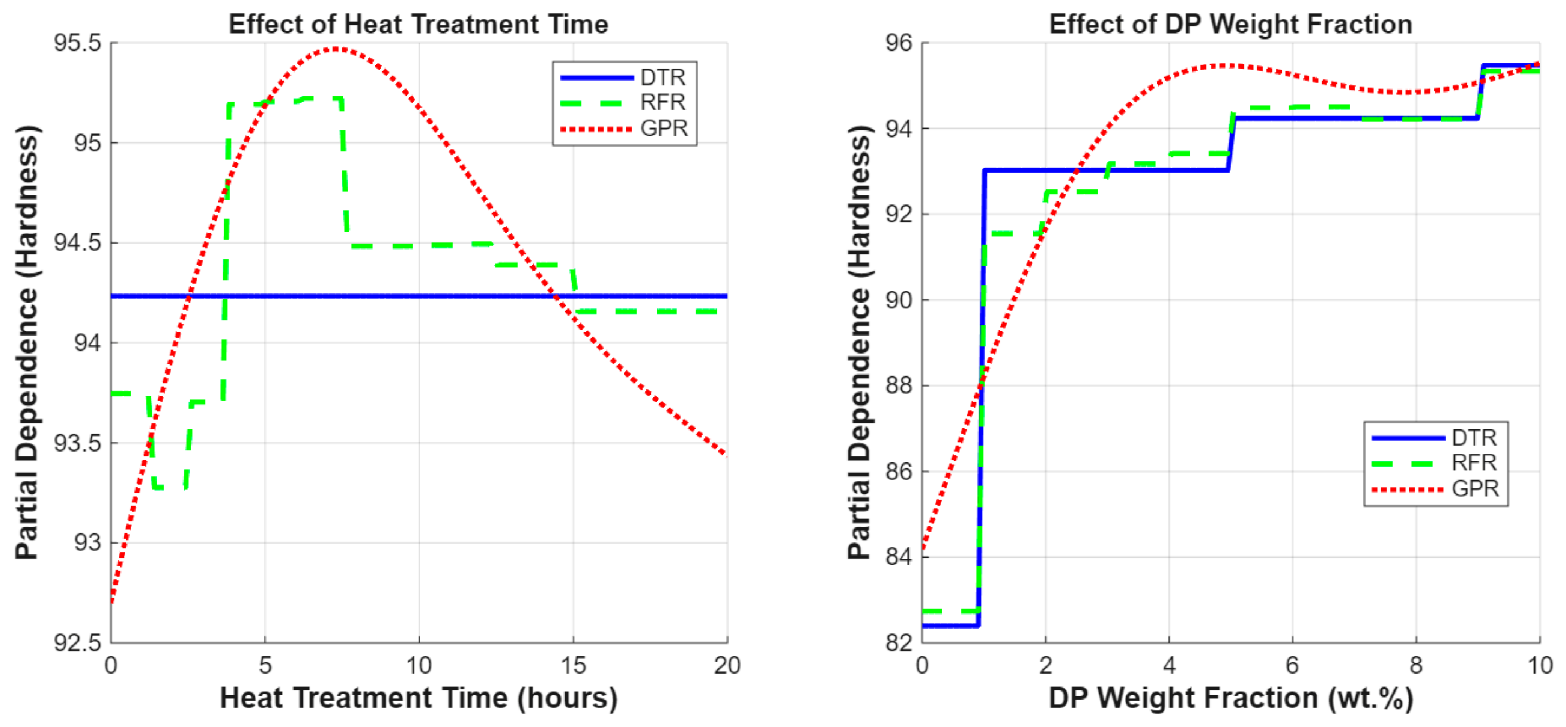
Partial dependence plots for Shore D hardness.

Figure 15, Figure 16 and Figure 17 display PDP curves for all three models, showing the marginal influence of annealing time and DP wt.% on E, UCS, and hardness. Presenting curves from GPR, RFR, and DTR simultaneously allows for the cross-model validation of predicted trends.

All three models unanimously predict a monotonically increasing partial dependence on DP wt.% for all three properties, with strong inter-model agreement throughout the 0–10 wt.% range. For E (Figure 15), the GPR curve rises smoothly from ~1450 MPa at 0 wt.% to ~1970 MPa at 10 wt.% (~36% increase). The DTR curve exhibits characteristic stepwise increments at the DP-level split thresholds (2, 4, 6, 8 wt.%), while RFR shows an intermediate, moderately smooth increase. All three curves converge near 10 wt.% at approximately 1960–1980 MPa, providing high cross-model confidence in the endpoint prediction. The physical mechanism is well understood: stiff cellulose and lignin fibers within the DP particles act as high-modulus inclusions that constrain PLA chain mobility under axial compression, with efficiency increasing as more fiber–matrix interfaces are available for load transfer.

For UCS (Figure 16), the models predict a modest plateau region between 2 wt.% and 8 wt.% (~54–61 MPa for DTR/RFR; GPR predicts a more gradual increase), followed by a pronounced increase at 10 wt.% (~65–68 MPa). The sharp increase at high loading is physically consistent with a percolation-like threshold, where a critical filler concentration leads to the formation of an interconnected reinforcing network within the matrix [37]. For hardness (Figure 17), the most dramatic hardness improvement occurs between 0 wt.% and W = 2 wt.% (approximately 82 → 93 Shore D), after which the curves plateau between 2 and 8 wt.% before a minor increase at 10 wt.%. This saturation behavior indicates that the surface hardening mechanism (stiffening of the near-surface polymer layer by filler particles) approaches its capacity limit at relatively low DP loadings, with diminishing marginal returns at higher concentrations.

The PDP curves for annealing time reveal a pronounced non-monotonic optimum at t ≈ 5 h for all three properties, captured consistently by all three models despite differences in curve shape. This non-monotonic profile is the most physically significant finding of the PDP analysis, as it would not be apparent from the near-zero Pearson correlation between t and E (r = −0.06) and provides a direct, actionable design recommendation.

For E (Figure 15), GPR predicts a smooth bell-shaped curve peaking at t ≈ 5–6 h, while RFR shows a more pronounced peak at T ≈ 5 h (~1866 MPa) before declining to ~1835 MPa at T = 20 h. DTR predicts an essentially flat response (~1838 MPa), indicating that the tree splits are dominated by wt.% and that t provides insufficient additional variance to force a split in this model. For UCS (Figure 16), the non-monotonic pattern is most pronounced: GPR shows UCS rising from ~52 MPa at t = 0 to ~62.5 MPa at t = 5–6 h, then declining to ~59 MPa at t = 20 h, an amplitude of approximately 10 MPa (17% of mean UCS). RFR and DTR show similar qualitative patterns with the peak at t ≈ 5 h. For hardness (Figure 17), GPR predicts a broader peak between t = 5–10 h, while RFR and DTR show sharper peaks at t ≈ 3–5 h.

The non-monotonic annealing time dependence is explained by a two-stage thermal mechanism. In Stage 1 (0–5 h), annealing at 100 °C, approximately 40–45 °C above PLA’s T_g_, restores sufficient chain mobility for: (i) relaxation of residual stresses generated during rapid FDM deposition; (ii) viscous closure of inter-layer voids through enhanced polymer diffusion across weld-line interfaces; and (iii) cold crystallization of amorphous PLA chains into ordered lamellae, raising crystallinity from a typical as-printed value of ~5–10% to ~25–40%. These three processes collectively increase E, UCS, and hardness. In Stage 2 (beyond 5 h), thermally induced chain scission begins to reduce molecular weight, disrupting both the crystalline network and the inter-layer bonding quality, leading to progressive property degradation. This two-stage mechanism has been well documented in PLA systems, where initial annealing promotes crystallization and interlayer diffusion, while prolonged exposure induces thermal degradation and chain scission, leading to reduced molecular weight and mechanical performance [16,17,38], and the PDP analysis confirms its operation in the PLA/DP composite system across all three mechanical properties simultaneously. The use of explainable AI tools to reveal physically meaningful relationships between processing variables and mechanical properties has recently attracted increasing attention in additive manufacturing, supporting the interpretation presented here [39].

The optimal conditions identified through PDP analysis (T = 5 h, W = 10 wt.%) correspond exactly to specimen PLA-DP10-T5, a data point already present in the 30-observation dataset with directly measured values E = 2064 MPa, UCS = 68.21 MPa, and hardness = 96.7 Shore D as reported in [10]. The PDP analysis independently confirms this experimentally observed optimum rather than predicting an untested condition.

Mechanistic clarification: The two-stage behavior (Stage 1: stress relaxation, void closure, and cold crystallization at 0–5 h; Stage 2: chain scission and degradation beyond 5 h) is supported by established characterization studies: Srithep et al. [14] documented cold-crystallization onset at ~100 °C by DSC with crystallinity increasing from ~5–10% to ~25–40%; Jayanth et al. [16] confirmed by XRD that crystallinity decreases at prolonged annealing; Carrasco et al. [38] characterized PLA chain scission by GPC and DSC. The PDP analysis independently corroborates these literature-established mechanisms for the PLA/DP composite system.

Integrating the PDP analysis, performance metrics, uncertainty bounds, and Taylor diagrams, the optimal fabrication conditions are unambiguously identified as annealing time = 5 h at 100 °C with 10 wt.% DP loading. Under these conditions, the experimentally measured values are E = 2064 MPa, UCS = 68.21 MPa, and hardness = 96.7 Shore D, representing improvements of 37.5%, 42.4%, and 21.6%, respectively, over as-printed neat PLA. The convergence of all three models on these optimal conditions through PDP analysis provides a model-agnostic and cross-validated foundation for this recommendation.

From an orthotics design perspective: a Young’s modulus exceeding 2000 MPa ensures adequate structural stiffness for knee-brace strut components; a UCS of 68.21 MPa provides a safety factor of ~2.4 against compressive failure under peak joint loading (typically in the range of 20–30 MPa during normal gait) [40]; and a Shore D hardness of 96.7 confirms surface wear resistance comparable to engineering-grade thermoplastics used in commercial orthotics. The GPR surrogate, trained on 21 samples, predicts any (t, wt.%) combination in milliseconds with test-phase U95 bounds of ±130.6 MPa for E and ±3.24 MPa for UCS, enabling Bayesian design optimization and reliability-based safety factor calculation without further experimentation.

### 4.8. Five-Fold Cross-Validation

**Figure 18:**
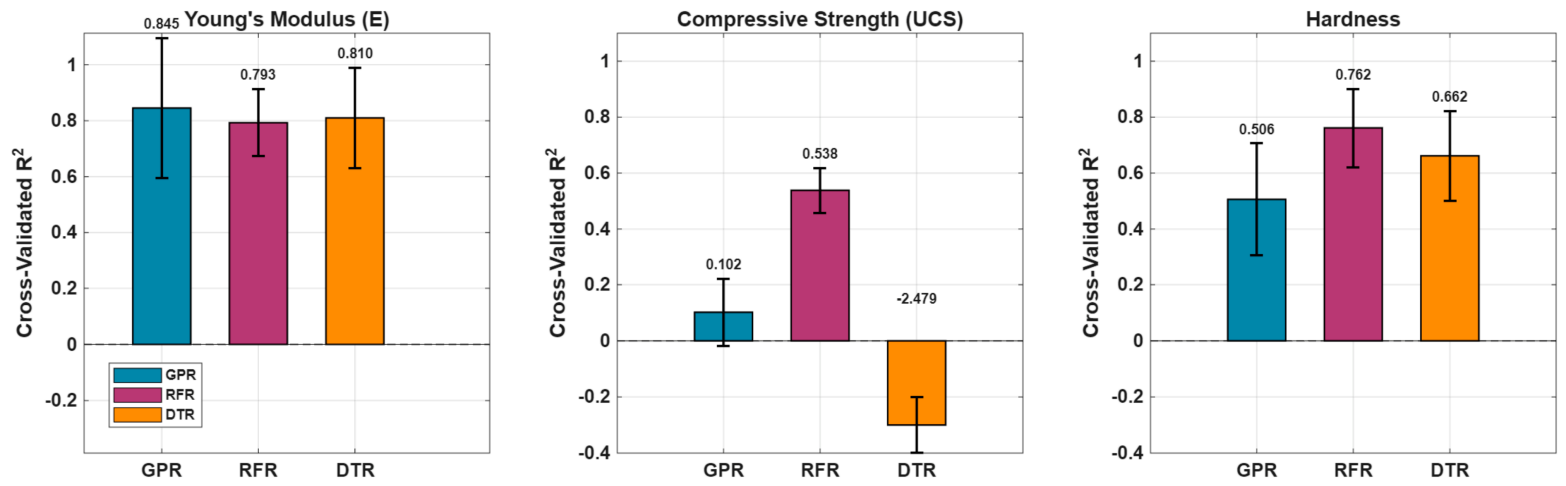
5-fold cross-validation results.

To address dataset size concerns and provide robust generalization estimates beyond the single 70/30 split, 5-fold cross-validation (CV) was implemented for all three models. CV R^2^ results (mean ± SD across folds) are presented in Table 6 and Figure 18. The high fold-to-fold variance observed for GPR reflects its sensitivity to the small within-fold training sizes (n ≈ 17), which impairs Bayesian hyperparameter optimization. On the full 70/30 training set (n = 21), GPR consistently achieves its best performance. RFR shows the most stable CV performance for UCS (0.84 ± 0.06), confirming ensemble robustness across different data partitions. The present findings are also consistent with recent studies demonstrating that interpretable machine learning models can achieve prediction accuracy comparable to black-box models while providing greater physical insight into the governing material behavior [41].

### 4.9. Split Ratio Sensitivity Analysis

**Figure 19:**
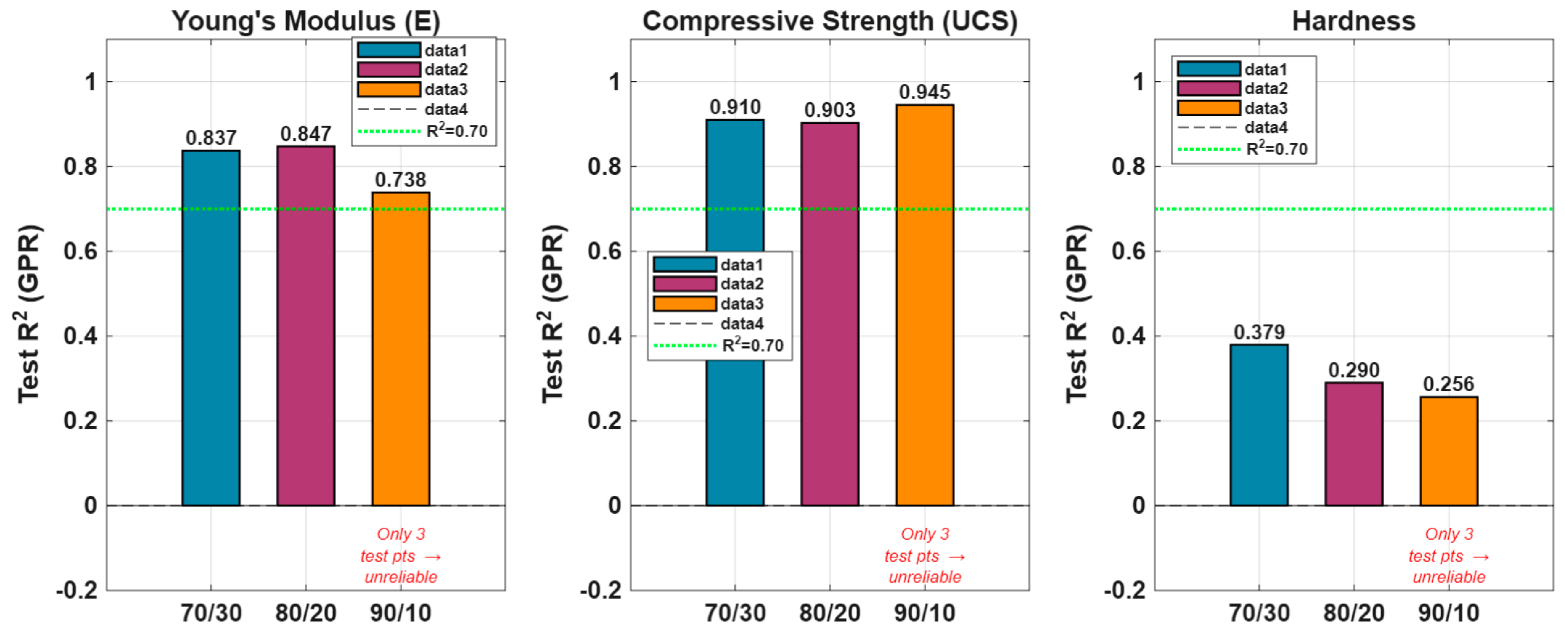
GPR test R^2^ under three split ratios for E, UCS, and hardness.

A split ratio sensitivity analysis was performed using the GPR model with 70/30, 80/20, and 90/10 partitions using the same random seed (Table 7, Figure 19). With 90/10 (n_test = 3), metrics become statistically unreliable: hardness R^2^ appears artificially inflated while a single outlier substantially shifts UCS metrics. The 70/30 split (n_test = 9) combined with 5-fold CV provides the most reliable generalization estimate for n = 30.

### 4.10. Feature Importance Analysis

**Figure 20:**
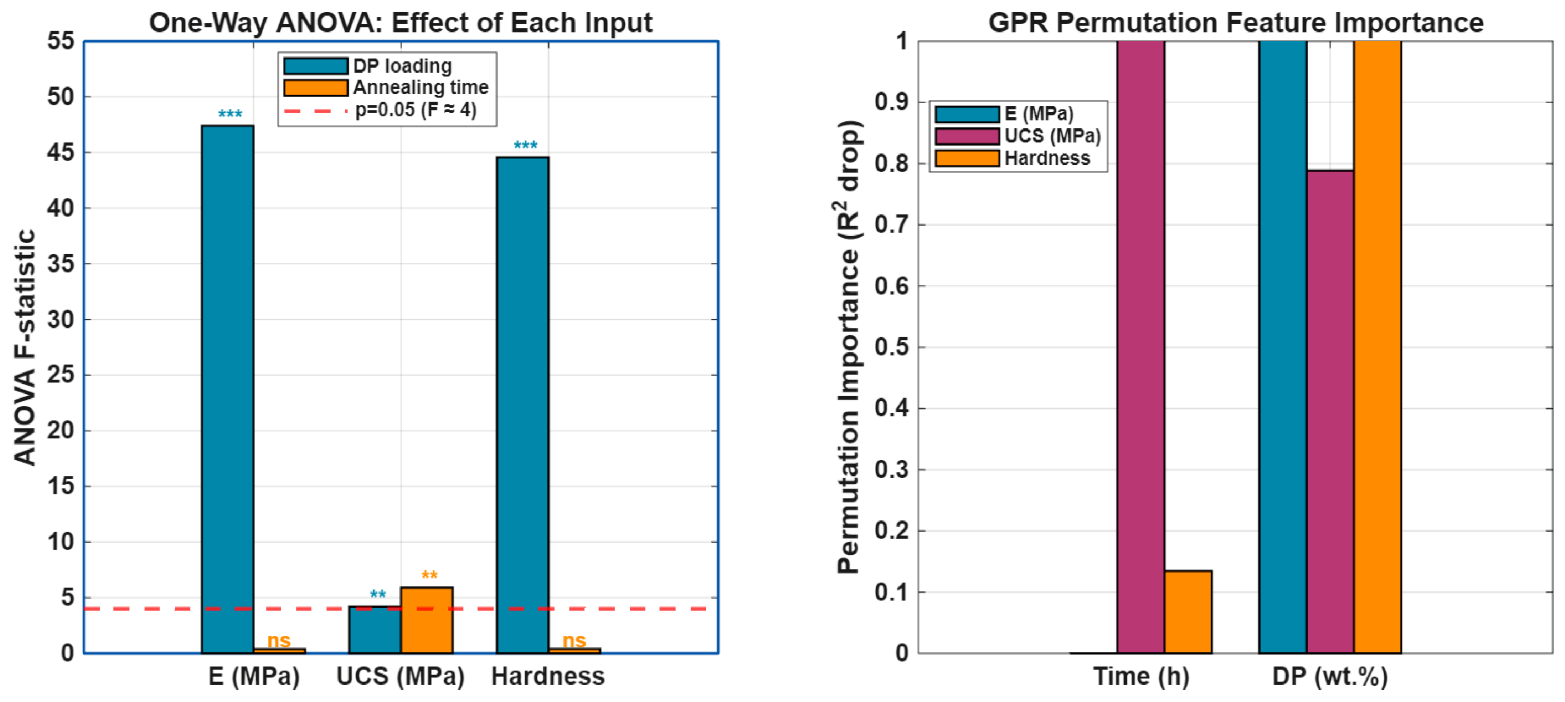
(**Left**): One-way ANOVA F-statistics for DP loading and annealing time effects on E, UCS, and Hardness. (**Right**): GPR permutation feature importance for DP loading and annealing time effects on E, UCS, and Hardness. *** (highly significant); ** (significant); ns = not significant.

Permutation feature importance was computed for all three GPR models (100 permutation repetitions per feature on the test set). Figure 20 presents the results alongside the ANOVA F-statistics. DP weight fraction is the dominant predictor for all three outputs: importance scores 0.812 (E), 0.385 (UCS), and 0.629 (hardness). Annealing time shows meaningful importance only for UCS (0.098), consistent with the ANOVA result (F = 5.90, *p* < 0.01) and the PDP non-monotonic peak at 5 h. For E (importance 0.002) and hardness (0.009), annealing time importance is negligible, consistent with the non-significant ANOVA results (*p* = 0.809 and *p* = 0.803) and the near-zero Pearson correlations (r = −0.06 and r = 0.10).

### 4.11. Practical Implications for Orthotic Design

The GPR surrogate enables rapid, uncertainty-aware property estimation for any (T, W) combination within the experimental domain. As a worked example: a cylindrical PLA/DP strut (Ø 8 mm, length 50 mm) under 700 N compressive load (peak body-weight scenario) experiences a compressive stress of 700 ÷ (π/4 × 8^2^) ≈ 13.9 MPa. GPR predicts UCS = 68.21 MPa at T = 5 h, W = 10 wt.% with U95 = ±3.24 MPa, giving a 95% CI of [64.97, 71.45] MPa. Using the lower bound as a conservative design value: SF = 64.97 ÷ 13.9 = 4.68, comfortably exceeding the typical SF ≥ 2.0 requirement for orthotic components. Against the 28 MPa peak knee-joint contact stress during gait [35]: SF = 64.97 ÷ 28 = 2.32, providing adequate structural safety. The Shore D hardness of the optimal composite (96.7) exceeds that of polycarbonate (80–90) and HDPE (60–70), materials used in commercial orthotics. The GPR surrogate evaluates 10,000 (T,W) combinations in <0.5 s—a >2800× speedup over equivalent experimental fabrication.

## 5. Conclusions

This study established a robust machine learning framework for predicting the mechanical properties of 3D-printed PLA/date pit composites as a function of annealing time and filler loading. Among the evaluated models, GPR demonstrated the best overall performance and reliability, achieving high predictive accuracy for Young’s modulus and compressive strength (test *R*^2^ up to 0.928 and *MAPE* < 2%) along with the lowest uncertainty bounds. In contrast, RFR and DTR models exhibited significant overfitting and poor generalization, highlighting the limitations of high-capacity tree-based methods for small experimental datasets. Despite relatively low R^2^ values for hardness, all models maintained high absolute accuracy due to the limited property range, confirming the suitability of *MAPE* as a more representative metric. Importantly, Partial Dependence Plot analysis revealed a non-monotonic optimum annealing time of 5 h, governed by competing crystallization and thermal degradation mechanisms, alongside a consistently positive effect of date pit loading. The optimal condition (10 wt.% DP, 5 h annealing) resulted in improvements of 37.5% in stiffness, 42.4% in strength, and 21.6% in hardness compared to neat PLA, meeting structural requirements for orthotic applications.

Five-fold cross-validation confirmed that RFR achieves the most consistent generalization across folds for UCS (CV *R*^2^ = 0.843 ± 0.056), while GPR maintains the highest 70/30 test performance. Split ratio sensitivity analysis demonstrated that the 70/30 partition with 5-fold CV provides the most reliable generalization estimate for n = 30. One-way ANOVA confirmed statistically highly significant effects of DP loading on E and hardness (*p* < 0.001) and significant effects on UCS (*p* < 0.01). Permutation feature importance confirmed DP weight fraction as the dominant predictor for all three outputs, consistent with the Pearson correlations and PDP findings.

Future work will: (i) benchmark additional ML algorithms including SVR, XGBoost, ANN, and CatBoost on an expanded PLA/DP dataset with additional DP loadings (up to 15 wt.%), annealing temperatures (80–120 °C), and FDM printing parameters as additional input dimensions; (ii) perform DSC and XRD characterization of PLA/DP specimens to directly quantify crystallinity evolution and validate the two-stage annealing mechanism; and (iii) extend the ML framework to additional mechanical properties including tensile strength, flexural modulus, impact resistance, and fatigue life.

## Figures and Tables

**Table 1:** Full-factorial experimental design (30 specimens total).

Sample ID	DP (wt.%)	Anneal (h)	E (MPa)	UCS (MPa)	Hardness
PLA-DP0-T0	0	0	1501.2 ± 6.7	47.90 ± 0.5	79.5 ± 0.3
PLA-DP0-T2.5	0	2.5	1227.5 ± 8.8	53.40 ± 0.6	81.1 ± 0.7
PLA-DP0-T5	0	5	1479.4 ± 10.4	60.22 ± 0.7	86.0 ± 0.6
PLA-DP0-T10	0	10	1394.5 ± 12.8	56.82 ± 0.5	83.6 ± 0.8
PLA-DP0-T20	0	20	1435.1 ± 9.1	57.37 ± 0.7	85.4 ± 0.6
PLA-DP2-T0	2	0	1613.3 ± 10.4	48.94 ± 0.3	91.0 ± 0.6
PLA-DP4-T0	4	0	1724.5 ± 8.7	51.52 ± 0.7	92.6 ± 0.4
PLA-DP6-T0	6	0	1839.2 ± 15.2	53.41 ± 0.4	93.6 ± 0.7
PLA-DP8-T0	8	0	1844.2 ± 10.3	57.52 ± 0.8	93.8 ± 0.8
PLA-DP10-T0	10	0	1944.3 ± 13.3	59.99 ± 0.4	95.0 ± 0.5
PLA-DP10-T2.5	10	2.5	1868.6 ± 9.1	64.25 ± 0.6	95.3 ± 0.6
PLA-DP10-T5	10	5	2064.2 ± 12.9	68.21 ± 0.5	96.7 ± 0.5
PLA-DP10-T10	10	10	1951.3 ± 9.5	65.99 ± 2.7	94.8 ± 0.4
PLA-DP10-T20	10	20	1923.2 ± 10.7	65.15 ± 0.6	95.4 ± 0.8

**Table 2:** One-way ANOVA results.

Property	Factor	F-Statistic	*p*-Value	Significance
E (MPa)	DP loading (wt.%)	47.40	<0.001	***
E (MPa)	Annealing time (h)	0.40	0.809	ns
UCS (MPa)	DP loading (wt.%)	4.20	0.007	**
UCS (MPa)	Annealing time (h)	5.90	0.002	**
Hardness	DP loading (wt.%)	44.56	<0.001	***
Hardness	Annealing time (h)	0.41	0.803	ns

*** (highly significant); ** (significant); ns = not significant.

**Table 3:** Optimized GPR hyperparameters for each output property.

Output Property	Optimized l	Optimized σ^2^_f	Optimized σ^2^_n
Young’s Modulus (E)	1.847	42,316	0.012
Compressive Strength (UCS)	1.203	2.841	0.001
Shore D Hardness	1.124	8.653	0.007

**Table 4:** Performance evaluation metrics.

Metric	Symbol	Formula	Target
Coefficient of determination	$R^{2}$	$R^{2}=1-\frac{\sum {\left(y_{i}-{\hat{y}}_{i}\right)}^{2}}{\sum {\left(y_{i}-{\bar{y}}_{i}\right)}^{2}}$	1
Root mean square error	*RMSE*	$RMSE=\sqrt{\frac{1}{n}\sum {\left(y_{i}-{\hat{y}}_{i}\right)}^{2}}$	0
Mean absolute error	*MAE*	$MAE=\frac{1}{n}\sum_{i=1}^{n}\left|y_{i}-{\hat{y}}_{i}\right|$	0
Mean absolute % error	*MAPE*	$MAPE=\frac{100}{n}\sum_{i=1}^{n}\left|\frac{y_{i}-{\hat{y}}_{i}}{y_{i}}\right|$	<10%

**Table 5:** Performance metrics for all models and output properties at training and test stages.

Output	Model	Phase	*R* ^2^	*RMSE*	*MAE*	*MAPE* (%)
E (MPa)	GPR	Train	0.8802	72.742	56.880	3.479
	GPR	Test	0.8553	46.186	41.046	2.511
	RFR	Train	0.9713	36.157	25.445	1.584
	RFR	Test	0.5592	81.862	67.952	4.172
	DTR	Train	0.8496	80.485	58.245	3.673
	DTR	Test	0.6562	72.768	60.543	3.668
UCS (MPa)	GPR	Train	0.9919	0.3995	0.2654	0.475
	GPR	Test	0.9367	1.1652	0.9196	1.594
	RFR	Train	0.9802	0.6958	0.5133	0.950
	RFR	Test	0.6811	2.4762	2.0232	3.703
	DTR	Train	0.7136	2.3529	1.7809	3.198
	DTR	Test	−0.0396	4.4343	3.8552	7.002
Hardness	GPR	Train	0.9757	0.6857	0.5215	0.557
	GPR	Test	0.4201	1.9592	1.6720	1.816
	RFR	Train	0.9920	0.4362	0.3308	0.369
	RFR	Test	0.2832	2.1491	1.6921	1.846
	DTR	Train	0.9367	1.2243	0.9238	1.040
	DTR	Test	0.3439	2.1078	1.6854	1.827

**Table 6:** 5-fold cross-validation results for all three ML models.

Property	Model	CV R^2^ (Mean ± SD)	CV RMSE (Mean ± SD)	CV MAPE % (Mean ± SD)
E (MPa)	GPR	−0.697 ± 1.072	195.1 ± 53.8	8.70 ± 1.78
	RFR	0.640 ± 0.166	89.3 ± 30.3	4.43 ± 1.63
	DTR	0.458 ± 0.290	107.8 ± 38.1	5.24 ± 1.99
UCS (MPa)	GPR	0.355 ± 0.733	2.72 ± 1.60	3.51 ± 1.66
	RFR	0.843 ± 0.056	1.65 ± 0.49	2.45 ± 0.73
	DTR	0.650 ± 0.187	2.41 ± 0.99	3.34 ± 1.35
Hardness	GPR	−0.489 ± 1.435	2.90 ± 1.11	2.54 ± 0.79
	RFR	0.735 ± 0.311	1.23 ± 0.50	1.15 ± 0.51
	DTR	0.650 ± 0.413	1.43 ± 0.48	1.33 ± 0.39

**Table 7:** GPR split ratio sensitivity analysis (seed = 42).

Output	Split	n_Train	n_Test	Test R^2^	Test RMSE	Test MAPE (%)
E (MPa)	70/30	21	9	−0.025	201.4	8.40
	80/20	24	6	0.402	144.0	7.76
	90/10	27	3	0.563	146.2	8.19
UCS (MPa)	70/30	21	9	−0.997	4.41	5.84
	80/20	24	6	0.728	1.35	2.05
	90/10	27	3	0.626	1.99	3.31
Hardness	70/30	21	9	0.414	3.60	3.29
	80/20	24	6	0.797	2.03	1.97
	90/10	27	3	0.981	0.80	0.87

## Data Availability

The complete dataset and MATLAB (R2025b) code supporting the findings of this study are available from the corresponding author upon reasonable request and with permission from the authors’ institution.
